# Clearance or Hijack: Universal Interplay Mechanisms Between Viruses and Host Autophagy From Plants to Animals

**DOI:** 10.3389/fcimb.2021.786348

**Published:** 2022-01-03

**Authors:** Wenxian Wu, Xiumei Luo, Maozhi Ren

**Affiliations:** ^1^ Institute of Urban Agriculture, Chinese Academy of Agricultural Sciences, Chengdu Agricultural Science and Technology Center, Chengdu, China; ^2^ Zhengzhou Research Base, State Key Laboratory of Cotton Biology, School of Agricultural Science of Zhengzhou University, Zhengzhou, China; ^3^ Hainan Yazhou Bay Seed Laboratory, Sanya, China; ^4^ Key Laboratory of Plant Hormones and Development Regulation of Chongqing, School of Life Sciences, Chongqing University, Chongqing, China

**Keywords:** virus–host interaction, immune response, autophagy, virus manipulation, infection

## Abstract

Viruses typically hijack the cellular machinery of their hosts for successful infection and replication, while the hosts protect themselves against viral invasion through a variety of defense responses, including autophagy, an evolutionarily ancient catabolic pathway conserved from plants to animals. Double-membrane vesicles called autophagosomes transport trapped viral cargo to lysosomes or vacuoles for degradation. However, during an ongoing evolutionary arms race, viruses have acquired a strong ability to disrupt or even exploit the autophagy machinery of their hosts for successful invasion. In this review, we analyze the universal role of autophagy in antiviral defenses in animals and plants and summarize how viruses evade host immune responses by disrupting and manipulating host autophagy. The review provides novel insights into the role of autophagy in virus–host interactions and offers potential targets for the prevention and control of viral infection in both plants and animals.

## Introduction

Plant and animal viruses are among the most difficult “foes” to deal with. Plant virus infections can lead to a substantial decrease in crop yield and represent a serious threat to food security. Global economic losses due to plant viruses are estimated to be as high as 30 billion dollars annually (Nicaise, 2014). The damage caused by mammalian viruses is even more widespread, as evidenced by the coronavirus disease 2019 (COVID-19) pandemic, with 200 million cases of infection and more than 4 million deaths being reported worldwide (Kumar et al., 2021). In addition, viruses such as human immunodeficiency virus (HIV), influenza virus, and hepatitis virus, among others, are endemic in humans and have long represented a threat to human health. Viruses are specialized parasites containing genomes with limited genome coding potential and, consequently, require the host’s intracellular machinery to replicate, express viral proteins, and establish infection (Cesarman et al., 2019; He et al., 2020; V’Kovski et al., 2021). In turn, hosts have evolved a variety of defense mechanisms to limit viral replication and spread. Plants have developed innate pathogen-associated molecular pattern (PAMP)-triggered immunity (PTI), effector-triggered immunity (ETI), RNA silencing, and a variety of other mechanisms to inhibit viral infection (Incarbone and Dunoyer, 2013; Mandadi and Scholthof, 2013). Similarly, animals control viral invasion by initiating innate and adaptive immune responses (Daugherty and Malik, 2012; Mukherjee, 2020). Interestingly, increasing evidence has shown that autophagic processes play an indispensable role in host immune responses in both plants and animals. Autophagy is also known to mediate a variety of host–virus interactions (Dong and Levine, 2013; Paul and Munz, 2016; Kushwaha et al., 2019; Leary et al., 2019).

Macroautophagy (hereinafter referred to as autophagy) is a conserved intracellular pathway in plants and animals through which cytosolic contents are encircled by specialized double-layered membrane vesicles, known as autophagosomes, that then transported them to lysosomes/vacuoles for degradation. Under normal conditions, autophagy functions primarily as a housekeeper in the maintenance of cellular homeostasis. Under conditions of stress such as starvation, aging, and microbial infections, autophagic activity is enhanced, and cellular homeostasis is maintained through the degradation of damaged cellular components in the cytoplasm, which, in turn, promotes cell and organism survival (Mizushima and Komatsu, 2011; Marshall and Vierstra, 2018). In animal cells, autophagy is involved in controlling viral infection not only by directly degrading viral components but also through regulating the intensity of the inflammatory response or promoting viral antigen presentation by major histocompatibility complexes (Choi et al., 2018). The role of autophagy in animal defenses against viral pathogens *in vivo* has been extensively studied; however, less is known about the function of autophagy in plant–virus interactions. During the long-term “arms race” between viruses and eukaryotes, autophagy is the “commanding height” that viruses must conquer, not only because it represents a cell-autonomous defense mechanism against microbial invasion, but also because it functions as a scaffold to promote viral replication. Autophagy also provides lipid membranes, and vectors for viral exit from cells, and can improve the survival rate of infected cells, thereby increasing the viral load (Lussignol and Esclatine, 2017; Wang et al., 2019; Keller et al., 2020). Indeed, many viruses have evolved a variety of strategies to disrupt or manipulate cellular autophagy to promote their own replication and spread.

Long-term mutual adaptation has resulted in an extremely complex interaction between hosts and viruses. In this review, we analyze the universal role of autophagy in antiviral defenses in animals and plants. We further discuss how viruses hijack host autophagic pathways to evade immune responses and promote self-replication and highlight a versatile virus–host autophagy interaction mechanism that exists in both plants and animals. This review provides novel insights into the role of autophagy in virus–host interactions and offers potential targets for the prevention and control of viral infection in both groups of eukaryotes.

## Autophagy Machinery

The core feature of the autophagy machinery is the formation of autophagosomes. This process is sequentially regulated by autophagy-related proteins (ATGs) that aggregate into complexes that hierarchically promote autophagy initiation, vesicle nucleation, phagosome expansion, cargo uptake, autophagosome closure, autophagosome–vacuoles/lysosome fusion, and content degradation (Dikic and Elazar, 2018; Ding et al., 2018). Many excellent reviews have focused on the molecular mechanism involved in autophagy (Chen and Klionsky, 2011; Michaeli et al., 2016; Dikic and Elazar, 2018; Ding et al., 2018; Soto-Burgos et al., 2018). In this section, we offer a context-dependent overview of autophagy, thus providing a basis for subsequent sections.

**Figure 1:**
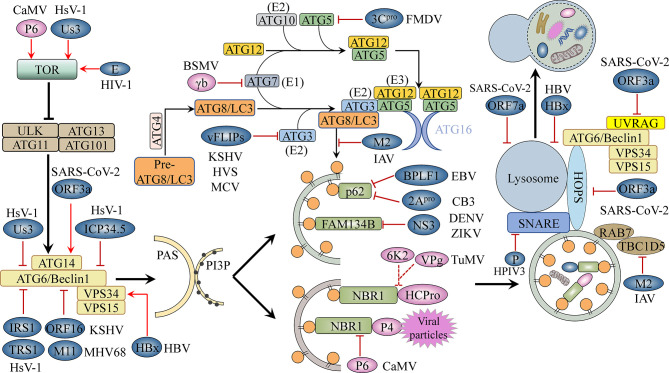
The autophagy machinery and its inhibition by viruses. The ATG1/ULK1 complex, the ATG6/Beclin1-PI3K/VPS34 complex, and the ATG12-ATG5-ATG16 and ATG8-PE conjugation systems, among others, are involved in key steps of the autophagy pathway, including initiation, elongation, completion, and fusion. Viral proteins block cellular autophagy and promote virus development by activating TOR, a conserved Ser/Thr kinase; interacting with autophagy-related proteins, thereby inhibiting or promoting their activity; targeting selective autophagy processes; and interfering with autophagosome–lysosome fusion or lysosomal acidification. Blue-grey ovals represent animal viral proteins. Plant viral proteins are shown in pink. TOR, target of rapamycin; PAS, pre-autophagosomal structure; PI3P, phosphatidylinositol-3-phosphate; E1/2/3, E1/2/3-like enzyme.

According to their function and physical interactions, core autophagy proteins can be divided into several functional units, namely, the ATG1/ULK1 (Unc-51-like kinase 1) complex; the ATG6/Beclin1-PI3K (phosphoinositide 3-kinase)/VPS34 (vacuolar protein sorting 34) complex; the ATG9 complex; the ATG12/ATG5/ATG16 ubiquitination-like conjunction system; and the ATG8/LC3 (microtubule-associated protein 1 light chain 3)-PE (phosphatidylethanolamine) ubiquitination-like conjunction system (Xie and Klionsky, 2007). The ATG1/ULK1 complex, consisting of the serine/threonine protein kinases ATG1/ULK1, ATG13, ATG101 and ATG11/FIP200 (FAK family-interacting protein of 200 kDa), is central to the initiation of autophagy (Hurley and Young, 2017). Multiple kinases upstream of autophagy can induce ATG1/ULK1 complex assembly and regulate autophagy initiation. For example, mammalian target of rapamycin kinase complex 1 (mTORC1) becomes inactivated once it senses stress associated with cellular energy and nutrient deprivation, resulting in the activation of the autophagy initiators ATG1/ULK1 and the promotion of the assembly of ATG1/ULK1, ATG13, and the accessory subunits ATG11 and ATG101 into an active complex (Hosokawa et al., 2009; Pu et al., 2017). This complex can stimulate several downstream phosphorylation-dependent autophagic steps, such as the delivery of lipids to constantly expanding phagocytic vesicles, as driven by the ATG9 complex composed of the transmembrane protein ATG9 as well as ATG2 and ATG18, which have been implicated in ATG9 recycling (Young et al., 2006; Zhuang et al., 2017). Another step, also phosphorylation-dependent, involves the activation of the ATG6/Beclin1-PI3K/VPS34 complex, which subsequently converts phosphatidylinositol (PI) from lipid molecules on the surface of phagocytic vesicles to phosphatidylinositol-3-phosphate (PI3P) (Russell et al., 2013). PI3P on isolation membranes is recognized by PI3P-binding factors (WD-repeat protein interacting with phosphoinositides [WIPIs, ATG18 homologous proteins]) that anchor to PI3P-decorated phagocytic vesicles and then recruit the ATG12-ATG5-ATG16 complex (Polson et al., 2010; Dooley et al., 2014; Proikas-Cezanne et al., 2015), which is composed of ATG12, ATG5, and ATG16 in a 2:2:2 ratio. Subsequently, ATG7 (E1-like enzyme) and ATG10 (E2-like enzyme) mediate the attachment of the C-terminal glycine of ATG12 to a conserved lysine residue within ATG5, yielding an ATG12-ATG5 conjugate, which then non-covalently binds to the dimeric scaffold protein ATG16 to form a hexameric complex with ligase activity (**Figure 1**) (Ichimura et al., 2000; Fujita et al., 2008). The ATG8-PE ubiquitination-like conjunction system plays an important role in the expansion of phagocytic vesicles, cargo uptake, and autophagosome closure (Nakatogawa et al., 2007). Inactive ATG8/LC3 protein is cleaved by the protease ATG4 to expose the conserved C-terminal glycine residue, and ATG7 (E1) transfers the cleaved ATG8 to ATG3 (E2). With the help of ATG12-ATG5-ATG16, acting as an E3 ligase, the C-terminal glycine carboxyl group of ATG8 covalently binds to the N-terminus of phosphatidylethanolamine components of the autophagy bilayer membrane, yielding lipidated ATG8/LC3, which mediates autophagic bilayer membrane expansion, closure, and autophagic cargo uptake (**Figure 1**) (Geng and Klionsky, 2008; Nakatogawa, 2013). Once the autophagosome is formed, it is transported to the vacuole/lysosome *via* a microtubule network controlled by the endosomal sorting complexes required for transport (ESCRT) machinery (Vietri et al., 2020). The autophagosome then fuses with the vacuole/lysosomal membrane, a process that is mediated by factors such as vesicular soluble N-ethylmaleimide-sensitive factor attachment protein receptor (v-SNARE) (Itakura et al., 2012).

Autophagy was initially thought to be a bulk catabolic process involving the re-mobilization of nutrients and the support of energy requirements, with cellular components being indiscriminately phagocytosed into the autophagosome. However, there is substantial evidence to indicate that autophagy can also degrade cytoplasmic cargo, such as misfolded/aggregated proteins, damaged organelles, and invading microorganisms, in a highly selective manner (Gatica et al., 2018; Marshall and Vierstra, 2018; Ran et al., 2020). ATG8/LC3 plays a key role in selective autophagy. Membrane-anchored ATG8/LC3 not only interacts with ATG1, ATG6/Beclin1, and other core autophagy proteins to synergistically regulate the initiation, extension, and maturation of autophagosomes, but also provides a platform for cargo receptors to selectively recruit their cargo (Johansen and Lamark, 2020). Autophagy cargo receptors typically recognize ubiquitinated substrates through a highly conserved ubiquitin-association domain (UBA) and anchor to the autophagosome *via* ATG8-interacting motifs (AIMs) (W/F/Y-x-x-L/I/W; also known as LC3-interacting regions [LIR] in animals), thereby recruiting cargo to the developing autophagosome for degradation (Johansen and Lamark, 2011; Birgisdottir et al., 2013). Notably, some autophagy receptors do not contain an AIM/LIR, but rather mediate selective cargo degradation through interaction with ATG8s *via* a ubiquitin interacting motif (UIM) (Marshall et al., 2019).

Selective autophagy can be subdivided into several types, including mitophagy [the removal of damaged or excessive mitochondria), reticulophagy (degradation of endoplasmic reticulum (ER)], aggrephagy (the degradation of protein aggregates), and proteaphagy (degradation of inactive proteasomes), among others (Gatica et al., 2018). In addition to maintaining cellular homeostasis, autophagy also participates in pathogen clearance. Selective autophagy of intracellular pathogens is called xenophagy, while that of virions or viral components is known as virophagy (Yla-Anttila, 2021). Selective autophagy depends on receptor recognition of cargo and the initiation of autophagosome formation (Huang et al., 2020; Vo and Choi, 2021). Substrate ubiquitination is often a key intermediary step in the recognition and degradation of these cargoes. For instance, in both plants and animals, once protein aggregates have been labeled through ubiquitination, the autophagy cargo receptor neighbor of brca 1 (NBR1) acts as a ubiquitin-binding protein that interacts with ubiquitinated protein aggregates and the core autophagy protein ATG8, following which both ubiquitinated proteins and NBR1 are degraded by autophagy (Kirkin et al., 2009; Jung et al., 2020).

## Autophagy-Mediated Antiviral Responses

Although studies have shown that autophagy plays distinct roles in host–virus interaction, corresponding to different viruses and host cell types, autophagy can be used to degrade viral components, viral particles, and even host factors required for viral replication; autophagy is therefore an important innate antiviral response (Choi et al., 2018; Ismayil et al., 2020; Yang et al., 2020). Many studies have confirmed that autophagy plays an antiviral defense role in host–virus interaction through silencing or mutating ATGs (Liu et al., 2005; Yordy et al., 2013).

### Autophagy as an Antiviral Strategy in Animal Cells

**Figure 2:**
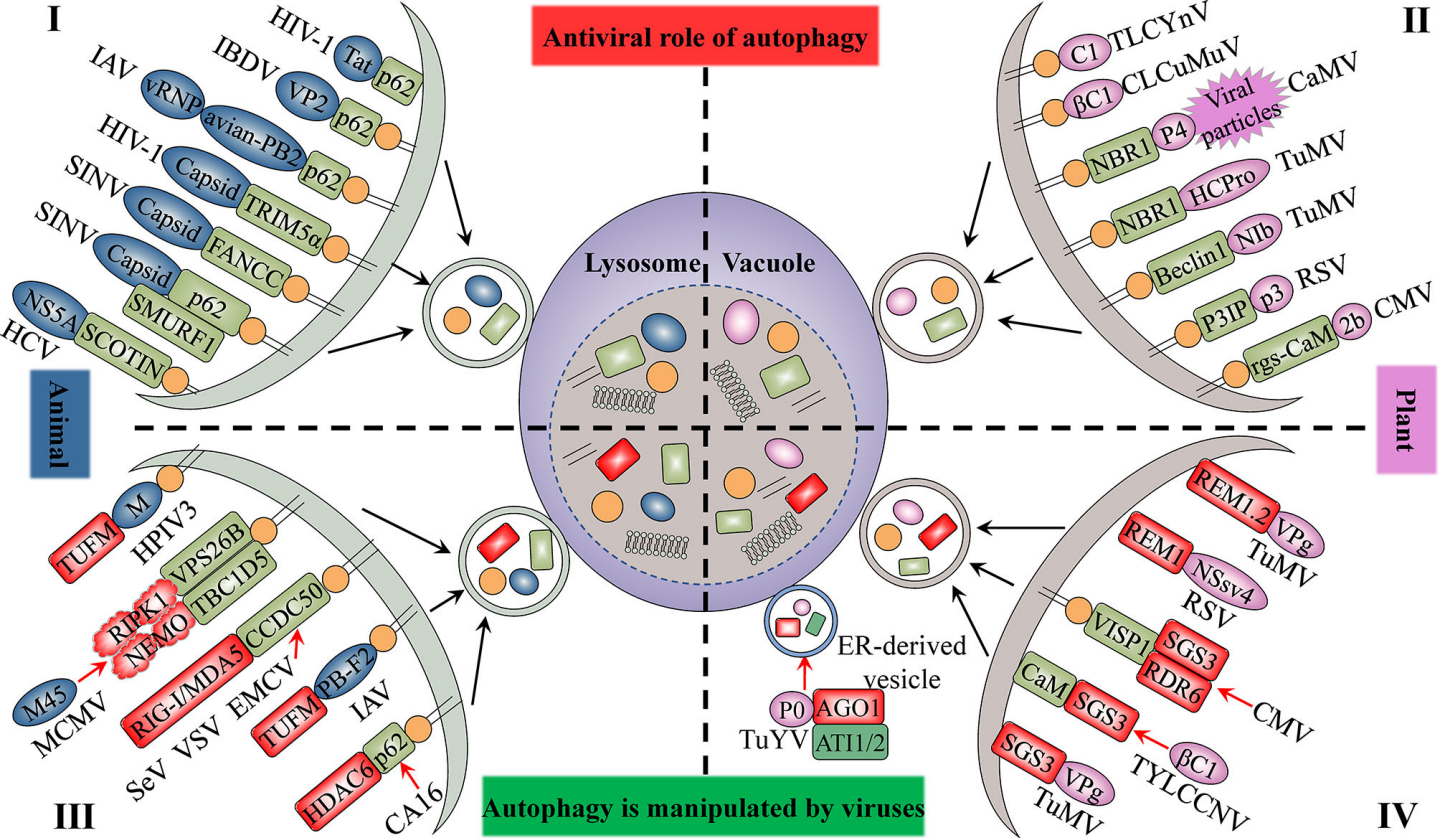
The antiviral role of autophagy and its manipulation by viruses. The upper panel indicates the antiviral aspects of cellular autophagy (I and II). Viral proteins that manipulate host autophagy are shown in the lower part (III and IV). The left (I and III) and right (II and IV) parts represent the interaction of animal viruses and plant viruses with host autophagy, respectively. Selective autophagy mediated by cargo receptors is an antiviral mechanism common to both animal and plant cells. Viruses derived from both plants and animals hijack autophagy to degrade factors that positively regulate host immune responses to enhance their self-proliferation. Blue-grey ovals represent animal viral proteins. Plant viral proteins are shown in pink. The grass-green rectangle with rounded corners represents host selective autophagy cargo receptors. Factors that positively regulate host immune responses are displayed in red.

The autophagy protein ATG5 is essential for protecting the mouse central nervous system from lethal infection with Sindbis virus (SINV). Orvedahl et al. (2010) reported that *Atg5* deletion resulted in the delayed clearance of viral proteins, while also leading to an increase in neuronal cell death and the cellular accumulation of the adaptor protein p62 [also known as sequestosome 1 (SQSTM1)]. The authors further found that p62 acts as a cargo receptor mediating the selective clearance of SINV capsid proteins, thereby promoting cell survival (**Figure 2**). High-throughput, genome-wide, small interfering RNA (siRNA) screening subsequently identified the host ubiquitin ligase SMURF1 as an essential factor for the colocalization of p62 and SINV capsid proteins as well as virophagy. Following the silencing of SMURF1, p62 lost its ability to target SINV (**Figure 2**) (Orvedahl et al., 2011). The Fanconi anemia complementation group C (FANCC) protein was also found to mediate virophagy by interacting with the SINV capsid and promoting host antiviral defenses (**Figure 2**) (Sumpter et al., 2016). SMURF1 and FANCC also target HSV-1 for selective degradation, suggesting that these two proteins generally function as virophagy factors (Orvedahl et al., 2011; Sumpter et al., 2016). A complex cell type- and infection status-dependent link exists between HIV-1 and autophagy. (Sagnier et al., 2015) reported that the HIV-1 reverse transcription activator Tat, a protein essential for viral transcription and virion production, is recognized by the adaptor protein p62/SQSTM1 in a ubiquitin-independent manner in CD^4+^ T-cells, following which it is degraded *via* selective autophagy (**Figure 2**). HIV-1 is a “master” at manipulating host cellular mechanisms, including autophagy, and facilitates its own replication and infection through disrupting or hijacking host cellular autophagic mechanisms. Influenza A virus (IAV) is an important zoonotic pathogen, causing significant morbidity in humans and representing an ever-present threat to humanity. IAV achieves efficient cross-species transmission through reassortment or directing host adaptation processes (Taubenberger and Kash, 2010). When IAV containing avian PB2 infects mammalian cells, viral ribonucleoprotein (vRNP) forms aggregates that localize to the microtubule-organizing center in infected cells (Liu et al., 2021). Correspondingly, the selective autophagy receptor p62/SQSTM1 targets newly synthesized vRNPs through PB2, a viral polymerase subunit, inducing higher autophagic flux and greater autolysosome accumulation, which limits viral infection (Liu et al., 2021). p62 can also mediate the degradation of avibirnavirus proteins. Infectious bursal disease virus (IBDV) capsid protein VP2 is responsible for virus assembly, maturation, and replication. (Li et al., 2020c) revealed that p62 recognizes ubiquitinated VP2 proteins and specifically recruits them to autophagosomes (**Figure 2**). p62 lacking the UBA or LIR can no longer promote VP2 degradation, indicating that p62 promotes the selective autophagic degradation of VP2 in a ubiquitin-dependent manner (Li et al., 2020c). It has been suggested that the ER-resident protein SCOTIN may act as a cargo receptor for virophagy and recruits non-structural 5A (NS5A), a key factor in hepatitis C virus (HCV) replication, into autophagosomes for degradation (**Figure 2**) (Kim et al., 2016). In animal cells, the antiviral restriction factors tripartite motif-containing proteins (TRIMs) comprise a large family of pattern recognition receptors (PRRs) containing a RING domain, a B box domain, and a coiled-coil domain at the N-terminus; additionally, most TRIMs contain a variable C-terminal domain that plays a role in substrate binding (Kawai and Akira, 2011). TRIMs not only regulate autophagy initiation and nucleation, but also act as cargo receptors to mediate the selective autophagy of viral capsid proteins. For example, in Langerhans cells (LCs), TRIM5α mediates the assembly of autophagy-activating complexes to turn on the virophagy machinery (Ribeiro et al., 2016). In addition, TRIM5α recruits the HIV-1 capsid into autophagosomes for HIV-1 degradation through directly interacting with the capsid and ATG8s (**Figure 2**) (Mandell et al., 2014a; Mandell et al., 2014b) (**Table 1**).

**Table 1:** Autophagy-mediated antiviral immune responses.

Host	Virus	Viral protein(s)	Host protein(s)	Functions	References
Animal	SINV	Capsid	p62; LC3	p62 adaptor protein mediates autophagic viral protein clearance, thus promoting cell survival	(Orvedahl et al., 2010)
	SINV	Capsid	SMURF1	Acts as a mediator of virophagy	(Orvedahl et al., 2011)
	SINV	Capsid	FANCC	Interacts with the capsid protein, facilitating virophagy	(Sumpter et al., 2016)
	HIV-1	Tat	p62	Selective degradation of Tat in a ubiquitin-independent manner	(Sagnier et al., 2015)
	IAV containing avian PB2	PB2; vRNP	p62; LC3	p62 targets vRNP to form an autophagosome through interaction with viral PB2	(Liu et al., 2021)
	IBDV	VP2	p62; LC3	p62 mediates the selective autophagic degradation of VP2, thus targeting IBDV replication	(Li et al., 2020b)
	HCV	NS5A	Scotin; LC3	Scotin recruits the NS5A protein to autophagosomes for degradation	(Kim et al., 2016)
	HIV-1	Capsid	TRIM5α; ATG8s	TRIM5α functions both as a regulator of autophagy and as an autophagic cargo receptor mediating HIV-1 restriction	(Mandell et al., 2014a; Mandell et al., 2014b; Ribeiro et al., 2016)
Plant	CLCuMuV	βC1	ATG8f	ATG8f targets βC1 for degradation	(Haxim et al., 2017)
	TLCYnV	C1	ATG8h; XPO1	ATG8h interacts with C1, directing it for degradation in an XPO1-mediated, nuclear export pathway-dependent manner	(Li et al., 2020a)
	CaMV	P4 and viral particles	NBR1; ATG8a	NBR1 targets P4 and viral particles, thus mediating their autophagy-dependent degradation	(Hafren et al., 2017)
	TuMV	HCPro	NBR1; ATG8a	HCPro is selectively degraded by the autophagy pathway through binding with NBR1	(Hafren et al., 2018)
	TuMV	NIb	Beclin1; ATG8a	Beclin1 interacts with Nib, targeting it for selective degradation	(Li et al., 2018)
	RSV	p3	P3IP; ATG8f	P3IP directs the selective autophagic degradation of p3 through interaction with ATG8, thereby limiting virus infection	(Jiang et al., 2021)
	CMV	2b	rgs-CaM; ATG8	rgs-CaM interacts with 2b for autophagy degradation	(Nakahara et al., 2012)

### Autophagy Plays a Role in Resistance to Plant Viruses

Several studies have provided clear evidence that autophagy can also serve as an antiviral defense in plants, directly targeting viruses or individual viral components for degradation. The cotton leaf curl Multan virus (CLCuMuV) βC1 protein is a key factor in virus-induced disease symptoms and virus accumulation in plants (Jia et al., 2016). Haxim et al. (2017) showed that ATG8 directly targets βC1 for degradation, thereby protecting plants against this geminivirus (**Figure 2**). Interestingly, the V32A mutation in βC1 disrupts the interaction between ATG8 and βC1, preventing βC1 degradation through autophagy. In *Nicotiana benthamiana*, CLCuMuV carrying the βC1^V32A^ mutation induces severe symptoms and viral DNA accumulation (Haxim et al., 2017). Similarly, tomato leaf curl Yunnan virus (TLCYnV) nucleoprotein C1 undergoes autophagic degradation through directly interacting with ATG8h (**Figure 2**). During virophagy, exportin1 (XPO1) participates in the transfer of C1 from the nucleus to the cytoplasm and mediates the binding of ATG8h to C1 (Li et al., 2020b). When the autophagy-related genes *ATG8h*, *ATG5*, and *ATG7* are separately knocked out in plants, the degradation of C1 is inhibited, thus promoting TLCYnV infection (Li et al., 2020b) (**Table 1**).

In plants, besides the direct targeting of the core autophagy protein ATG8 to the viral component, virophagy also requires cargo receptors as intermediaries. Hafren et al. (2017) reported that selective autophagy limits cauliflower mosaic virus (CaMV) infection during compatible interactions between CaMV and host plants. Autophagy-defective Arabidopsis mutants (*atg5* and *atg7*) develop more severe symptoms after CaMV infection than their wild-type counterparts. The viral capsid protein P4 specifically accumulates in *Atg5* and *Atg7* mutant strains, whereas the levels of other viral proteins remain unchanged. Furthermore, selective autophagy mediated by the cargo receptor NBR1 inhibits the accumulation of CaMV P4 (**Figure 2**) (Hafren et al., 2017). NBR1-dependent selective autophagy has also been reported to function as an antiviral mechanism targeting RNA viruses. NBR1 inhibits turnip mosaic virus (TuMV) accumulation by targeting the TuMV helper component protease HCpro, a suppressor of antiviral RNA silencing (**Figure 2**) (Hafren et al., 2018). Nevertheless, TuMV appears to antagonize NBR1-dependent autophagy during infection through the activity of different viral proteins, thus limiting its antiviral ability (see below). ATG6/Beclin1 reportedly acts as a selective autophagic cargo receptor and interacts with RNA-dependent RNA polymerase (RdRp) by targeting its GDD motif, which results in the autophagic degradation of RdRp and the inhibition of TuMV infection (**Figure 2**) (Li et al., 2018). Silencing ATG6/Beclin1 or ATG8 to block autophagy can promote RdRp accumulation and viral infection and *vice versa*. That the GDD motif is relatively conserved and is found in the RdRps of most plant and animal viruses suggests that ATG6/Beclin1 may be a general cargo receptor in virophagic processes (Li et al., 2018). Recently, Jiang et al. (2021) identified a novel cargo receptor, P3IP, which induces and mediates the autophagic degradation of the rice stripe virus (RSV)-encoded RNA silencing suppressor (RSS) P3 protein (**Figure 2**). The calmodulin-like protein rgs-CaM may also be a selective autophagic receptor that regulates viral infection. For example, tomato rgs-CaM can interact with and stimulate the autophagic degradation of a variety of viral RNA RSSs, including HCpro from potyvirus as well as the 2b protein from cucumber mosaic virus (CMV) and tomato aspermy virus (**Figure 2**) (Nakahara et al., 2012) (**Table 1**).

In summary, selective autophagy mediated by cargo receptors has been shown to play a key role in host protection against viral infection. Meanwhile, this antiviral mechanism is well conserved in animal and plant cells. The identification of receptors or adaptors for selective autophagy will greatly advance the understanding of virophagy and better reveal the biological processes underlying the role of selective autophagy in host–virus interactions.

## Autophagy is Subverted by Viruses

In response to the limiting effects of autophagy on viral infection, persistent viruses have developed both specific and non-specific strategies to inhibit or disrupt multiple steps of the autophagy pathway for effective replication (**Table 2**) (Choi et al., 2018; Huang et al., 2020).

**Table 2:** Autophagy is subverted by viruses.

Host	Virus(s)	Viral protein(s)	Host protein(s)	Effects on host–virus interactions	References
Animal	HIV-1	Envelope	mTORC1	The envelope protein activates the mTORC1 pathway, leading to autophagy exhaustion	(Blanchet et al., 2010)
	HSV-1	Us3	mTORC1	Us3 activates mTORC1, which inhibits the ULK autophagy-promoting complex	(Rubio and Mohr, 2019)
	HSV-1	Us3	Beclin1	Us3 associates with and phosphorylates Beclin1, thus limiting autophagy and promoting virus replication	(Rubio and Mohr, 2019)
	HSV-1	ICP34.5	Beclin1	ICP34.5 interacts with Beclin1, thus inhibiting autophagy	(Orvedahl et al., 2007)
	HCMV	TRS1	Beclin1	TRS1 interacts with Beclin1, thus inhibiting autophagy	(Chaumorcel et al., 2012)
	HCMV	IRS1	Beclin1	IRS1 blocks host autophagy by interacting with Beclin1	(Mouna et al., 2016)
	KSHV	ORF16	Beclin1	ORF16 mimics cellular Bcl-2 and attenuates autophagy through direct interaction with Beclin1	(Pattingre et al., 2005)
	MHV68	M11	Beclin1	M11 mimics cellular Bcl-2 and attenuates autophagy through direct interaction with Beclin1	(Pattingre et al., 2005)
	KSHV; HVS; MCV	vFLIPs	ATG3	vFLIPs suppresses autophagy by preventing ATG3 from binding and processing LC3	(Lee et al., 2009)
	FMDV	3C^pro^	ATG5-ATG12	3C^pro^ suppresses autophagy *via* the degradation of the ATG5-ATG12 conjugate	(Fan et al., 2017)
	IAV	M2	LC3	M2 interacts with LC3 and promotes its relocalization to the host’s plasma membrane	(Beale et al., 2014)
	DENV; ZIKV	NS3	FAM134B	NS3 cleaves the FAM134B receptor, thereby suppressing the reticulophagy pathway	(Lennemann and Coyne, 2017)
	CB3	2A^pro^	p62	2A^pro^ cleaves p62, resulting in disrupted selective autophagy	(Shi et al., 2013; Mohamud et al., 2019)
	EBV	BPLF1	p62	BPLF1 targets p62 and decreases its ubiquitination, thus inhibiting selective autophagy	(Yla-Anttila et al., 2021)
	SARS-CoV-2	ORF3a	VPS39	ORF3a interacts with VPS39 and prevents the assembly of the SNARE complex	(Hayn et al., 2021; Koepke et al., 2021; Miao et al., 2021)
	SARS-CoV-2	ORF7a	Unknown	ORF7a interferes with autophagosome acidification	(Hayn et al., 2021; Koepke et al., 2021)
	SARS-CoV-2	ORF3a	UVRAG	ORF3a interacts with UVRAG to inhibit PI3KC3-C2 and promote the formation of PI3KC3-C1	(Qu et al., 2021)
	HPIV3	P	SNAP29	P binds to SNAP29 and prevents SNARE proteins from mediating autophagosome–lysosome fusion	(Ding et al., 2014)
	IAV	M2	TBC1D5	M2 abrogates TBC1D5-Rab7 binding through interaction with TBC1D5	(Martin-Sancho et al., 2021)
	HBV	HBx	Unknown	HBx impairs lysosomal acidification	(Liu et al., 2014)
	PV	Unknown	Galectin 8; PLA2G16	Galectin 8 initiates the autophagic degradation of viral RNA, the virus uses PLA2G16 to evade galectin 8-mediated detection	(Staring et al., 2017)
Plant	CaMV	P6	TOR	P6 activates TOR kinase, which blocks cellular autophagy and promotes CaMV translation	(Zvereva et al., 2016)
	BSMV	γb	ATG7	γb interacts with ATG7 and disrupts ATG7-ATG8 interaction, thus suppressing autophagy and promoting viral infection	(Yang et al., 2018)
	CaMV	P6	NBR1	P6 disrupts the interaction between P4 and host NBR1, which protects viral replication factory inclusions from autophagic degradation	(Hafren et al., 2017)
	TuMV	VPg; 6K2	Unknown	VPg and 6K2 antagonize the antiviral capacity of NBR1-dependent autophagy by blocking NBR1 and HCpro degradation	(Hafren et al., 2018)
	RSV	NSvc4	Type-I J-domain proteins	NSvc4 hijacks UPR-activated type-I J-domain proteins, thus preventing its autophagic degradation	(Li et al., 2021)

### Viruses Disrupt Autophagosome Initiation and Nucleation

The TOR kinase complex functions upstream of autophagy, controlling autophagosome biogenesis by negatively regulating the activity of the ATG1/ULK1 complex (**Figure 1**). Some animal and plant viruses can activate TOR kinase activity to inhibit virophagy (**Table 2**). For example, CaMV, a plant virus, binds to and activates TOR kinase through the multifunctional protein P6, which, in turn, blocks cellular autophagy and promotes CaMV infection (**Figure 1**) (Zvereva et al., 2016). Regarding animal viruses, Blanchet et al. (2010) reported that HIV-1 envelope activates the mTORC1 pathway in dendritic cells (DCs), leading to impaired autophagy and, consequently, the blocking of autophagosome-mediated degradation (**Figure 1**). Autophagy is a powerful inhibitor of herpes simplex virus 1 (HSV-1) pathogenesis in neurons (Yordy et al., 2012). However, HSV-1 serine/threonine kinase Us3 can antagonize autophagy in non-neuronal cells by activating mTORC1 activity and increasing the phosphorylation level of ATG1/ULK1 (**Figure 1**) (Rubio and Mohr, 2019). Us3 can also directly phosphorylate ATG6/Beclin1 and inhibit its activity, which further suppresses cellular autophagy (**Figure 1**) (Rubio and Mohr, 2019). Indeed, ATG6/Beclin1 is a target of many animal viral proteins (**Table 2**). The HSV-1-encoded neurotoxic protein ICP34.5 binds to ATG6/Beclin1 and inhibits its autophagic function (**Figure 1**) (Orvedahl et al., 2007). The HSV-1 ICP34.5 mutant lacking the ATG6/Beclin1 binding domain cannot inhibit autophagy in neurons or cause fatal encephalitis in mice (Orvedahl et al., 2007). Human cytomegalovirus (HCM1) expresses TRS1, a functional homolog of ICP34.5, which can also block autophagosome biogenesis (**Figure 1**) (Chaumorcel et al., 2012). The N-terminal domain of TRS1 contains the Beclin1 binding region, which is crucial for inhibiting Beclin1-mediated autophagy (Chaumorcel et al., 2012). Another HCMV protein, IRS1, has also been reported to block autophagy by interacting with Beclin1 (**Figure 1**) (Mouna et al., 2016).

In yeast and mammalian cells, in addition to its anti-apoptotic role, B-cell lymphoma 2 (Bcl-2) also exerts anti-autophagic activity through its interaction with Beclin1 (Pattingre et al., 2005). Some animal viral proteins function in a manner similar to that of host cell Bcl-2, i.e., they attenuate autophagy by directly interacting with Beclin1 (**Table 2**). For example, Kaposi’s sarcoma-associated herpesvirus (KSHV) and murine gammaherpesvirus 68 (MHV68) encode the Bcl-2 paralogs ORF16 and M11, respectively. These viral Bcl-2 (vBcl-2) proteins mimic their cellular counterparts (cBcl-2) and inhibit autophagosome formation (**Figure 1**) (Pattingre et al., 2005). Furthermore, structural and biochemical analysis has shown that vBcl-2 has a significantly higher affinity for Beclin1 and inhibits autophagosome formation to a greater extent than cBcl-2 (Ku et al., 2008). Because of the lack of a regulatory loop that can undergo phosphorylation by c-Jun N-terminal kinase (JNK), vBcl-2 can remain bound to Beclin1, indicating that vBcl-2 has evolved into a highly effective autophagy inhibitor (Wei et al., 2008). Whether plant viruses can inhibit the activity of ATG6/Beclin1 through phosphorylation, direct interaction, or any other mechanisms, thereby blocking autophagosome biogenesis, is unknown. Moreover, no homolog of yeast and mammalian Bcl-2 has been identified in plants, and it is unclear whether a mechanism similar to the Bcl-2-mediated regulation of the autophagy pathway exists in plants. More studies investigating the regulation of host autophagy by plant viral proteins are needed to elucidate these possibilities.

### Viruses Disrupt Phagosome Expansion

The ATG12-ATG5-ATG16 and ATG8/LC3-PE ubiquitin-like conjugation systems are essential for autophagosome formation and may also be a driving force for vesicle membrane deformation or bending. Some animal and plant viral proteins bind to certain proteins of these two sets of ubiquitin-binding systems, thus interfering with autophagosome biogenesis (**Table 2**). The FLICE-like inhibitor protein (vFLIP) of KSHV, herpesvirus saimiri (HVS), and molluscum contagiosum virus (MCV) can inhibit autophagy by preventing ATG3 from binding and processing LC3 (**Figure 1**) (Lee et al., 2009). Foot-and-mouth disease virus (FMDV) mediates the degradation of the ATG5-ATG12 complex through viral 3C^pro^ (**Figure 1**) (Fan et al., 2017). The siRNA-mediated knockdown of ATG5-ATG12 significantly increases the FMDV load and *vice versa* (Fan et al., 2017). Plant viruses also adopt similar strategies to inhibit virophagy. For example, the γb protein of barley stripe mosaic virus (BSMV) interferes with the interaction between ATG7 and ATG8 in a competitive manner and disrupts autophagy-mediated antiviral defenses (**Figure 1**) (Yang et al., 2018). IAV targets ATG8/LC3, the core component of autophagy, through the multifunctional protein M2, thereby disrupting autophagy. Beale et al. (2014) found that the cytoplasmic tail of IAV M2 contains a highly conserved LIR that mediates a direct interaction between M2 and LC3 in virus-infected cells and thereby promoting the mislocalization of LC3 to the plasma membrane. Moreover, mutations in M2 abolish LC3 binding, interfere with virus budding, and reduce the stability of virus particles.

### Viruses Interfere With Selective Autophagy

Interference with cargo receptor-dependent selective autophagy is a commonly used strategy by animal and plant viruses to counteract host antiviral responses (**Table 2**). Some arboviruses, such as dengue virus (DENV) and Zika virus (ZIKV), use the ER as a source of membranes to establish replicative organelles and promote their assembly and final maturation along the secretory pathway (Welsch et al., 2009). Correspondingly, host cells have evolved reticulophagy to cope with this stress. For example, the ER-localized cargo receptor FAM134B can limit the replication of DENV and ZIKV (Khaminets et al., 2015). However, these virus-encoded NS3 proteases specifically block reticulophagy by cleaving FAM134B at a single site in its reticular homology domain (RHD) (**Figure 1**) (Lennemann and Coyne, 2017). p62/SQSTM1 plays an important role in mediating virophagy. For instance, p62/SQSTM1 can directly interact with the coxsackievirus B3 (CVB3) capsid protein VP1 and recruit it for autophagic degradation, which reduces intracellular viral protein production (Shi et al., 2013). Interestingly, CVB3 viruses use viral protease 2A^pro^ to cleave p62/SQSTM1, disrupting its function in selective autophagy (**Figure 1**) (Shi et al., 2013; Mohamud et al., 2019). In contrast, Epstein–Barr virus (EBV) targets p62/SQSTM1 *via* the deubiquitinase BPLF1, which inhibits selective autophagy and promotes EBV replication and transmission (**Figure 1**) (Yla-Anttila et al., 2021). Plant viruses employ a similar strategy to inhibit selective autophagy. For example, NBR1 targets the viral capsid protein P4 and mediates its autophagic degradation, which inhibits CaMV infection (Hafren et al., 2017). However, the P6 protein of CaMV can disrupt the interaction between host NBR1 and P4 and protect the inclusion bodies of viral replication factories from autophagic degradation (**Figure 1**) (Hafren et al., 2017). In addition, TuMV VPg and 6K2 can reportedly antagonize the antiviral ability of NBR1-dependent autophagy by blocking the degradation of HCpro by NBR1; however, the molecular mechanism underlying this inhibition of autophagy remains unclear (**Figure 1**) (Hafren et al., 2018).

### Viruses Block Autophagosome–Lysosome Fusion

In addition to activating the TOR kinase complex, inhibiting autophagic core protein activity, and disrupting selective autophagy, animal viruses can also interfere with autophagosome maturation and block autophagosome–lysosome fusion, thus suppressing antiviral autophagic activity (**Figure 1**). SARS-CoV-2 restricts autophagy-associated signaling and blocks autophagic flux. Cells infected with SARS-CoV-2 show an accumulation of key metabolites, the activation of autophagy inhibitors such as AKT and SKP2, and a reduction in the levels of several proteins responsible for processes spanning from autophagosome formation to autophagosome–lysosome fusion (Gassen et al., 2021). In a recent study, the effect of individual SARS-COV-2 proteins on autophagy was systematically analyzed, and the authors found that E, M, ORF3a, and ORF7a promoted autophagosome accumulation, while also reducing autophagic flux (Hayn et al., 2021; Koepke et al., 2021). Additionally, ORF3a and ORF7a were reported to block autophagy by respectively interfering with autophagosome–lysosome fusion and lysosomal acidification (Hayn et al., 2021; Koepke et al., 2021). Miao et al., (2021) conducted an in-depth analysis of the mechanism by which ORF3a prevents autophagosome–lysosome fusion. ORF3a strongly interacts with VPS39, a component of the tethering factor HOPS (homotypic fusion and protein sorting) complex. The binding of ORF3a to VPS39 disrupts the assembly of the HOPS complex, which is followed by the failure of STX17-SNAP29-VAMP8 SNARE complex assembly. As the SNARE complex mediates autophagosome–lysosome fusion, this effect of ORF3a leads to the inhibition of autophagosome–lysosome fusion and the complete blockage of autophagic flux (Miao et al., 2021; Yim and Mizushima, 2021). Qu et al. (2021) showed that ORF3a has another effect associated with host autophagy. The core autophagy protein ATG6/Beclin1 is known to regulate lipid kinase Vps34 (PI3KC3) and to interact with mammalian ATG14 or UVRAG to form two phosphatidylinositol 3-kinase complexes with significantly distinct functions. The PI3KC3-C1 (ATG6/Beclin1-Vps34-Atg14) complex positively regulates autophagosome formation while the PI3KC3-C2 complex (ATG6/Beclin1-Vps34-UVRAG) mediates autophagosome maturation by promoting autophagosome–lysosome fusion (Levine et al., 2015). ORF3a interacts with the autophagy regulator UVRAG to selectively inhibit PI3KC3-C2 and promote the formation of PI3KC3-C1, thus inducing incomplete autophagy (Qu et al., 2021). It is not clear whether SARS-CoV-2 protein is normally targeted for autophagic degradation. If so, blocking fusion will allow SARS-CoV-2 to avoid lysosomal degradation and prevent the degradation products from being used for antigen presentation to T cells. If not, the accumulation of membrane-related components caused by incomplete autophagy may exert a positive effect on SARS-CoV-2 replication. Interestingly, several other intractable viruses adopt similar strategies to avoid autophagosome–lysosome fusion. Human parainfluenza virus type 3 (HPIV3) phosphoprotein (P) binds to SNAP29 and inhibits its interaction with STX17, thus preventing autophagosome–lysosome fusion mediated by these two host SNARE proteins (Ding et al., 2014). IAV utilizes M2 to block fusion, resulting in autophagosome accumulation. M2 physically interacts with TBC1D5 through its cytoplasmic tail, thereby abrogating TBC1D5-Rab7 binding, which is critical for autophagosome–lysosome fusion (Martin-Sancho et al., 2021). Hepatitis B virus (HBV) is one of the most successful human pathogens. Liu et al. (2014) showed that the HBV X protein (HBx) significantly impairs lysosomal acidification and affects lysosomal maturation, thereby inhibiting autophagic degradation. However, HBx can also bind to and enhance the enzymatic activity of PI3KC3, an enzyme vital for initiating autophagy, which, in turn, promotes autophagosome formation in infected cells (Sir et al., 2010). Consequently, inducing incomplete autophagy may allow HBV to both avoid autophagic degradation and promote its own replication through making use of the components of the autophagy machinery.

There are no reports to date of plant viruses interfering with the mechanism of autophagy similar to that seen with animal viruses; however, given that plant viruses may promote replication through autophagy biogenesis, and because they must avoid degradation *via* the autophagy pathway, plant viruses may “kill two birds with one stone” by blocking the last link in the autophagic process, that is, the autophagosome maturation and fusion steps.

Utilizing host proteins to evade being degraded by autophagy is a versatile mechanism adopted by viruses. Galectin-8 can detect nucleosomes containing picornaviruses (PVs) and mark them for autophagic degradation; meanwhile, PVs such as poliovirus can evade this detection with the aid of the host protein HRAS-like suppressor 3 (PLA2G16), thus evading clearance by autophagy and ensuring the delivery of viral genomes into the cytoplasm (**Table 2**) (Staring et al., 2017). Rice streak virus (RSV) can induce an unfolded protein response (UPR) in both rice and tobacco. In turn, RSV-induced UPR activates the host’s autophagy pathway, targeting the RSV-encoded motor protein NSvc4 for autophagic degradation and inhibiting RSV movement between cells. Correspondingly, RSV NSvc4 hijacks UPR-activated type I J-domain proteins in plants to evade autophagic degradation (**Table 2**) (Li et al., 2021).

## Autophagy is Manipulated by Viruses

### Animal Viruses Hijack Autophagy, Leading to Weakened Immunity

Besides directly disrupting host autophagy, some plant- or animal-derived viruses can also hijack host cell autophagy, which leads to weakened host antiviral defense responses (**Table 3**). Recently, Hou et al. (2021) found that CCDC50 negatively regulates the type I interferon (IFN) signaling pathway that is activated by animal RNA viral sensor RIG-I-like receptors (RLRs). Interestingly, in human monocytes (THP-1) infected with RNA viruses such as Sendai virus (SeV), vesicular stomatitis virus (VSV), or encephalomyocarditis virus (EMCV), CCDC50 expression is significantly enhanced, and CCDC50 specifically recognizes polyubiquitinated RLRs, resulting in the delivery of activated RIG-I/MDA5 into autophagosomes for degradation (**Figure 2**) (Hou et al., 2021). Histone deacetylase 6 (HDAC6), a component of viral RNA-induced stress granules, acts as an antiviral immune complex and plays an active role in the type I IFN responses (Zheng et al., 2020). Coxsackievirus A16 (CA16) triggers p62-mediated selective autophagic degradation of HDAC6, inhibits type I IFN responses, and promotes viral replication (**Figure 2**) (Zheng et al., 2020). Some viral proteins act directly as cargo receptors and manipulate selective autophagy to inhibit host antiviral responses. For example, human parainfluenza virus type 3 (HPIV3) matrix protein (M) translocates to host mitochondria and induces mitophagy through interacting with Tu translation elongation factor, mitochondrial (TUFM) and the autophagy protein LC3 (**Figure 2**) (Ding et al., 2017). M-mediated mitophagy leads to the inhibition of type I IFN responses. The IAV PB1-F2 protein functions in a similar manner to HPIV3 M, simultaneously interacting with TUFM and LC3B to induce complete mitochondrial autophagy, which promotes the degradation of mitochondrial antiviral signaling protein (MAVS) and suppresses the host’s innate immunity (Wang et al., 2021a). Muscolino et al. (2020) detailed a mechanism by which viruses hijack cellular autophagy to degrade host signaling proteins and thus evade immunity. The M45 protein of murine cytomegalovirus induces the degradation of nuclear factor κ-light-chain-enhancer of activated B-cells (NF-κB) essential modulator (NEMO) and receptor-nuclear protein kinase 1 (RIPK1) by first promoting their sequestration as insoluble protein aggregates and then recruiting the retromer component vacuolar protein sorting 26B (VPS26B) and the LC3-interacting adaptor protein TBC1D5 to promote the degradation of the aggregates through selective autophagy (**Figure 2**) (Muscolino et al., 2020). Like M45, the HSV-1 ICP6 protein also induces the aggregation and degradation of RIPK1 (Muscolino et al., 2020).

**Table 3:** Autophagy is manipulated by viruses.

Host	Virus(s)	Viral protein(s)	Host protein(s)	Effects on host–virus interactions	References
Animal	SeV; VSV; EMCV	Unknown	CCDC50; RIG-I/MDA5	Enhances CCDC50 expression, which delivers activated RIG-I/MDA5 for autophagic degradation	(Hou et al., 2021)
	CA16	Unknown	p62; HDAC6	Triggers p62-mediated selective autophagic degradation of HDAC6	(Zheng et al., 2020)
	HPIV3	M	LC3; TUFM	M mediates mitophagy *via* interactions with TUFM and inhibits the type I interferon response	(Ding et al., 2017)
	IAV	PB1-F2	LC3; TUFM	PB1-F2 interacts with TUFM and LC3B, thus inducing complete mitochondrial autophagy	(Wang et al., 2021a)
	MCMV	M45	VPS26B; TBC1D5; NEMO; RIPK1	M45 promotes NEMO and RIPK1 aggregation and recruits VPS26B and TBC1D5 to facilitate the degradation of the aggregates through selective autophagy	Muscolino et al., 2020)
	HBV	SHBs	LC3	SHBs interacts with LC3 and induces autophagy *via* triggering UPR and ER stress	(Li et al., 2011)
Plant	TuYV	P0	AGO1; ATI1/2	P0 triggers AGO1 degradation by the autophagy pathway	(Derrien et al., 2012; Michaeli et al., 2019)
	TuMV	VPg	SGS3	VPg mediates the degradation of SGS3 by autophagy and ubiquitination	(Cheng and Wang, 2017)
	TYLCCNB	βC1	CaM; SGS3	βC1 upregulates CaM expression and promotes CaM-mediated SGS3 degradation	(Li et al., 2014; Li et al., 2017)
	CMV	Unknown	VISP1; SGS3/RDR6	CMV induces VISP1 expression, VISP1 interacts with SGS3 and mediates the autophagic degradation of SGS3/RDR6	(Tong et al., 2021)
	RSV	NSsv4	REM1	NSsv4 interacts with REM1 and interferes with its S-acylation, inducing the autophagic degradation of unmodified REM1	(Fu et al., 2018)
	TuMV	VPg	REM1.2	VPg interacts with REM1.2 and mediates REM1.2 degradation through autophagy and ubiquitination pathways	(Cheng et al., 2020)
	TuMV	6K2; NIb	NBR1; ATG8f	TuMV activates UPR-dependent NBR1-ATG8f autophagy to target the VRC to the tonoplast, thus promoting viral replication	(Li et al., 2020a)

### Plant Viruses Manipulate Autophagy to Counteract Host Antiviral Defenses

siRNA-mediated post-transcriptional gene silencing (PTGS) is a well-characterized conserved antiviral defense mechanism in higher plants. Key components involved in the PTGS mechanism include ARGONAUTE1 (AGO1) and the host RNA-dependent RNA polymerase 6 (RDR6)/suppressor of gene silencing 3 (SGS3) complex (Ding, 2010). During interaction with their hosts, plant viruses target these proteins for autophagic clearance to counteract host-mediated RNA silencing. Derrien et al. (2012) found that polerovirus P0 triggers AGO1 degradation through the autophagy pathway (**Figure 2**). Subsequently, Michaeli et al. (2019) showed that P0 and AGO1 are associated with the ER, leading to their loading into ER-associated vesicles and, subsequently, their vacuolar degradation in an ATG5- and ATG7-dependent manner. In addition, ATG8-interacting proteins 1 and 2 (ATI1 and ATI2) are recruited to the ER and interact with AGO1 to promote the ER-associated autophagic degradation of AGO1 (Michaeli et al., 2019). TuMV infection has been reported to reduce the level of SGS3, which is essential for the biosynthesis of virus-derived small interfering RNA (vsiRNA) (Cheng and Wang, 2017). TuMV-encoded viral genomic connexin (VPg) interacts with SGS3 and induces its degradation and that of its interacting partner RDR6 through both 20S ubiquitin-proteasome and autophagic pathways (**Figure 2**) (Cheng and Wang, 2017). Li and co-workers reported that geminiviruses appear to indirectly utilize the plant endogenous RNA silencing suppressor calmodulin-like protein (CaM) to inhibit the siRNA mechanism by promoting the autophagic degradation of SGS3 (**Figure 2**) (Li et al., 2014; Li et al., 2017). Recently, Tong et al. (2021) identified a novel 71-amino acid virus-induced small peptide (VISP1) in plants that acts as an autophagy cargo receptor. VISP1 overexpression induces selective autophagy, which attenuates SGS3/RDR6-dependent viral siRNA amplification and enhances viral infection; meanwhile, VISP1 mutants display the opposite effect. Some plant viruses can induce the upregulation of VISP1 expression, thus mediating selective autophagic degradation of SGS3/RDR6 and, consequently, promoting self-replication and infection (**Figure 2**).

Remorins (REMs) are plant-specific membrane-associated proteins that play an important role in the interaction between plants and pathogens (Konrad et al., 2014). Fu et al. (2018) reported that S-acylation is a prerequisite for NbREM1 to target plasma membrane microdomains and is also required for its antiviral function, i.e., the inhibition of intercellular virus transport. Meanwhile, the RSV motor protein NSvc4 interacts with NbREM1 and interferes with its S-acylation; accumulated (untargeted) NbREM1 is degraded by autophagy, leading to the downregulation of NbREM1. In summary, RSV attenuates NbREM1-mediated antiviral activity, which promotes viral infection (**Figure 2**) (Fu et al., 2018). Similarly, Cheng et al. (2020) reported that TuMV VPg interacts with REM1.2 and mediates its degradation through the 26S ubiquitin-proteasome and autophagy pathways (**Figure 2**).

### Viruses Exploit the Autophagy Machinery for Replication

Viruses can also directly use the autophagy machinery to promote their own replication. For example, TuMV upregulates NBR1-mediated selective autophagy in a UPR-dependent manner and targets viral RdRp-containing virus replication complex (VRC) to the vacuolar membrane, which promotes viral replication and virion accumulation through cascades of protein–protein interactions (**Table 3**) (Li et al., 2020a). Animal viruses also induce autophagosome accumulation by activating the UPR; however, they do not promote the degradation of autophagic proteins (Sir et al., 2008). Importantly, this autophagosome accumulation enhances HCV replication, suggesting that HCV uses an incomplete autophagic response to promote its replication (Sir et al., 2008). The production and envelopment of another animal virus, HBV, is also dependent on the host autophagy machinery. Unsurprisingly, HBV enhances the autophagy process in host cells without promoting protein degradation. This enhancement is mediated by HBV small surface protein (SHBs), which induces autophagy *via* triggering UPR and ER stress (Li et al., 2011).

In summary, plant and animal viruses employ several strategies to manipulate the autophagy machinery of their hosts. Viruses promote antiviral factor degradation by activating selective autophagy receptors or inducing the expression of negative immune regulators in their hosts. Additionally, virus-encoded proteins act as selective autophagy receptors and mediate autophagy, thus obstructing antiviral responses. Finally, some viruses use their host’s autophagic process directly, but do not promote autophagic protein degradation to enhance viral replication.

## Conclusions and Perspectives

The past decade has seen significant progress in the study of autophagy–virus interactions. It is well-established that this ancient and conserved catabolic pathway is a key element of antiviral immunity *via* mediating the selective elimination of viral proteins and particles. However, in the long-term “arms race” with their hosts, viruses have evolved a variety of strategies to inhibit and disrupt the autophagy pathway, thereby limiting the hosts’ antiviral ability, and even manipulate and use autophagy to enhance infection. Here, we have reviewed the research progress related to the interaction between autophagy and viruses, and summarized the process of virophagy mediated by a variety of selective autophagy receptors. Moreover, we have described the similar strategies used by plant and animal viruses, including the autophagy-induced activation of negative regulatory signals, direct inhibition of key proteins involved in autophagosome biogenesis, interruption of selective autophagy, and use of host proteins to evade autophagic degradation, all of which disrupt antiviral responses. In addition, we compared the mechanisms used by plant and animal viruses to manipulate autophagy and promote self-replication and infection, highlighting that both plant and animal viruses can manipulate selective receptors in the host or produce proteins that act as cargo receptors, thus inhibiting antiviral immune responses.

Although numerous studies have investigated the control of viral infection by autophagy and how viruses counteract autophagy-induced adverse consequences, autophagy–virus interactions remain ill-defined. Additionally, the mechanisms involved in how viral material is specifically recognized and targeted for degradation *via* autophagy remain poorly understood. A key direction for future research will be to identify and characterize virophagy receptors that drive host defense responses as well as the role played by ubiquitination and/or other post-translational modifications in selectivity and cargo recognition. These will greatly improve our knowledge of the mechanisms and functions of autophagy in plant immunity. Additionally, an interesting balance exists in some viruses, especially RNA viruses, in that because they can neither survive nor escape autophagy, they have evolved a mechanism that blocks only some aspects of autophagy. For example, herpesviruses can effectively prevent autophagosome maturation; in turn, autophagosomes represent a source of their outer membrane. It remains unclear how the virus subtly regulates autophag signaling during its infection cycle such that it can simultaneously escape autophagic degradation while exploiting the structural benefits provided by autophagy.

Compared with plant viruses, substantially more is known about interactions between animal viruses and host autophagy. Some directions for research on plant autophagy–virus interactions can be garnished from our knowledge of animal viruses, which can be summarized as follows: (1) In animal cells, autophagy controls viral infection at three levels. At the first level, autophagy directly mediates the selective degradation of viral components or particles; the second level involves the autophagy-mediated initiation of the innate immune response through synergizing with pattern recognition receptor signaling to induce IFN production; at the third level, meanwhile, autophagy activates adaptive immunity by promoting antigen presentation. Although innate and adaptive immune response mechanisms do not exist in plant cells, factors such as hormones, PTI, and ETI play key roles in plant antiviral responses. Studies have shown that autophagy is associated with the salicylic acid signaling pathway and programmed cell death; however, the intrinsic crosstalk mechanisms have not been explored. In addition, it is not known whether the complex regulatory mechanism, including negative feedback, found between autophagy and immune receptors in animals also exists in plants. Yang et al. (2019) reported that autophagy is involved in the degradation of the plant immune receptor FLS2, suggesting that such a mechanism does indeed exist in plants. Revealing the relationship between autophagy and plant immune signaling will greatly increase our knowledge of the mechanisms and functions of autophagy in plant immunity and antiviral responses. (2) Accumulating evidence has suggested that within animal cells, many components of the autophagy machinery also mediate autophagy-independent antiviral functions (Galluzzi and Green, 2019). For example, ATG16L1-dependent targeting of LC3 to single-membrane, non-autophagosome compartments—referred to as non-canonical autophagy—protects mice from lethal IAV infection (Wang et al., 2021b). However, whether ATGs in plant cells also have autophagy-independent functions remains largely unknown. (3) There is a unique virophagy pathway controlled by SNX5 in animal cells. This pathway can activate the autophagy-related PI3KC3-C1 kinase complex and produce the key autophagy initiation signal PI3P, thus activating autophagy (Dong et al., 2021). Snx5-regulated virophagy has no effect on basic autophagy and autophagy induced by multiple classical or non-classical stimuli, and is not related to other cellular pathways, including endocytosis and interferon signaling, but rather to a specific viral defense responses through autophagy (Dong et al., 2021).

It is not known whether plants have a similar virophagy-specific machinery. (4) Many animal viruses encode proteins that target ATG6/Beclin1 and inhibit its activity, thus blocking autophagosome nucleation and maturation. This mechanism of disrupting autophagosome biosynthesis has not been found in plant viruses. However, given the dual role of ATG6/Beclin1 in mediating antiviral responses, ATG6/Beclin1 should be an ideal target for plant viruses. (5) Animal viruses can also interfere with the maturation of host autophagosomes and their fusion with lysosomes, achieving the effect of “killing two birds with one stone”, whereby they promote their own replication while also avoiding degradation *via* the autophagy pathway. No such reports exist regarding plant viruses. Further in-depth investigation of the autophagic mechanism in plants and the strategies used by plant viruses against autophagy may shed light on these questions. (6) The molecular basis underlying virus-induced autophagy activation in plants and the role of plant autophagy proteins and membranes in viral replication, which have been established in animal systems, is currently unknown. In summary, further investigation is required to reveal the specific role of autophagy in antiviral infection as well as the mechanism by which viruses manipulate autophagy.

## Author Contributions

WW and XL prepared the original draft. WW and XL undertook editing. MR was involved in supervision. XL and MR acquired the funding. All authors contributed to the article and approved the submitted version.

## Funding

This work was funded by the National Key R&D Program of China (2017YFE0115500), the National Natural Science Foundation of China (31801911), the Basic Research and Frontier Exploration Foundation of Chongqing (cstc2018jcyjAX0753), the Central Public-Interest Scientific Institution Basal Research Fund (Y2021XK05), and the Science and Technology Innovation Project of the Chinese Academy of Agricultural Sciences (No. 34- IUA- 02).

## Conflict of Interest

The authors declare that the research was conducted in the absence of any commercial or financial relationships that could be construed as a potential conflict of interest.

## Publisher’s Note

All claims expressed in this article are solely those of the authors and do not necessarily represent those of their affiliated organizations, or those of the publisher, the editors and the reviewers. Any product that may be evaluated in this article, or claim that may be made by its manufacturer, is not guaranteed or endorsed by the publisher.

## References

[B1] Beale R. Wise H. Stuart A. Ravenhill B. J. Digard P. Randow F. (2014). A LC3-Interacting Motif in the Influenza A Virus M2 Protein is Required to Subvert Autophagy and Maintain Virion Stability. Cell Host Microbe 15 (2), 239–247. doi: 10.1016/j.chom.2014.01.006 24528869 PMC3991421

[B2] Birgisdottir A. B. Lamark T. Johansen T. (2013). The LIR Motif - Crucial for Selective Autophagy. J. Cell Sci. 126 (Pt 15), 3237–3247. doi: 10.1242/jcs.126128 23908376

[B3] Blanchet F. P. Moris A. Nikolic D. S. Lehmann M. Cardinaud S. Stalder R.. (2010). Human Immunodeficiency Virus-1 Inhibition of Immunoamphisomes in Dendritic Cells Impairs Early Innate and Adaptive Immune Responses. Immunity 32 (5), 654–669. doi: 10.1016/j.immuni.2010.04.011 20451412 PMC2929482

[B4] Cesarman E. Damania B. Krown S. E. Martin J. Bower M. Whitby D. (2019). Kaposi Sarcoma. Nat. Rev. Dis. Primers 5 (1), 9. doi: 10.1038/s41572-019-0060-9 30705286 PMC6685213

[B5] Chaumorcel M. Lussignol M. Mouna L. Cavignac Y. Fahie K. Cotte-Laffitte J.. (2012). The Human Cytomegalovirus Protein TRS1 Inhibits Autophagy. via its interact Beclin 1. J. Virol. 86 (5), 2571–2584. doi: 10.1128/JVI.05746-11 PMC3302257 22205736

[B6] Cheng X. Wang A. (2017). The Potyvirus Silencing Suppressor Protein VPg Mediates Degradation of SGS3 *via* Ubiquitination and Autophagy Pathways. J. Virol. 91 (1), e01478-16. doi: 10.1128/JVI.01478-16 27795417 PMC5165207

[B7] Cheng G. Yang Z. Zhang H. Zhang J. Xu J. (2020). Remorin Interacting With PCaP1 Impairs Turnip Mosaic Virus Intercellular Movement But is Antagonised by VPg. New Phytol. 225 (5), 2122–2139. doi: 10.1111/nph.16285 31657467

[B8] Chen Y. Klionsky D. J. (2011). The Regulation of Autophagy - Unanswered Questions. J. Cell Sci. 124 (Pt 2), 161–170. doi: 10.1242/jcs.064576 21187343 PMC3037094

[B9] Choi Y. Bowman J. W. Jung J. U. (2018). Autophagy During Viral Infection - a Double-Edged Sword. Nat. Rev. Microbiol. 16 (6), 341–354. doi: 10.1038/s41579-018-0003-6 29556036 PMC6907743

[B10] Daugherty M. D. Malik H. S. (2012). Rules of Engagement: Molecular Insights From Host-Virus Arms Races. Annu. Rev. Genet. 46, 677–700. doi: 10.1146/annurev-genet-110711-155522 23145935

[B11] Derrien B. Baumberger N. Schepetilnikov M. Viotti C. De Cillia J. Ziegler-Graff V.. (2012). Degradation of the Antiviral Component ARGONAUTE1 by the Autophagy Pathway. Proc. Natl. Acad. Sci. U.S.A. 109 (39), 15942–15946. doi: 10.1073/pnas.1209487109 23019378 PMC3465452

[B12] Dikic I. Elazar Z. (2018). Mechanism and Medical Implications of Mammalian Autophagy. Nat. Rev. Mol. Cell Biol. 19 (6), 349–364. doi: 10.1038/s41580-018-0003-4 29618831

[B13] Ding S. W. (2010). RNA-Based Antiviral Immunity. Nat. Rev. Immunol. 10 (9), 632–644. doi: 10.1038/nri2824 20706278

[B14] Ding B. Zhang L. Li Z. Zhong Y. Tang Q. Qin Y.. (2017). The Matrix Protein of Human Parainfluenza Virus Type 3 Induces Mitophagy That Suppresses Interferon Responses. Cell Host Microbe 21 538-547 (4), e534. doi: 10.1016/j.chom.2017.03.004 28407488

[B15] Ding X. Zhang X. Otegui M. S. (2018). Plant Autophagy: New Flavors on the Menu. Curr. Opin. Plant Biol. 46, 113–121. doi: 10.1016/j.pbi.2018.09.004 30267997

[B16] Ding B. Zhang G. Yang X. Zhang S. Chen L. Yan Q.. (2014). Phosphoprotein of Human Parainfluenza Virus Type 3 Blocks Autophagosome-Lysosome Fusion to Increase Virus Production. Cell Host Microbe 15 (5), 564–577. doi: 10.1016/j.chom.2014.04.004 24832451

[B17] Dong X. Levine B. (2013). Autophagy and Viruses: Adversaries or Allies? J. Innate Immun. 5 (5), 480–493. doi: 10.1159/000346388 23391695 PMC3790331

[B18] Dong X. Yang Y. Zou Z. Zhao Y. Ci B. Zhong L.. (2021). Sorting Nexin 5 Mediates Virus-Induced Autophagy and Immunity. Nature 589 (7842), 456–461. doi: 10.1038/s41586-020-03056-z 33328639 PMC7856200

[B19] Dooley H. C. Razi M. Polson H. E. Girardin S. E. Wilson M. I. Tooze S. A. (2014). WIPI2 Links LC3 Conjugation With PI3P, Autophagosome Formation, and Pathogen Clearance by Recruiting Atg12-5-16l1. Mol. Cell 55 (2), 238–252. doi: 10.1016/j.molcel.2014.05.021 24954904 PMC4104028

[B20] Fan X. Han S. Yan D. Gao Y. Wei Y. Liu X.. (2017). Foot-And-Mouth Disease Virus Infection Suppresses Autophagy and NF-Small Ka, CyrillicB Antiviral Responses. via degrad ATG5-ATG12 by 3C(pro). Cell Death Dis. 8 (1), e2561. doi: 10.1038/cddis.2016.489 PMC5386389 28102839

[B21] Fujita N. Itoh T. Omori H. Fukuda M. Noda T. Yoshimori T. (2008). The Atg16L Complex Specifies the Site of LC3 Lipidation for Membrane Biogenesis in Autophagy. Mol. Biol. Cell 19 (5), 2092–2100. doi: 10.1091/mbc.E07-12-1257 18321988 PMC2366860

[B22] Fu S. Xu Y. Li C. Li Y. Wu J. Zhou X. (2018). Rice Stripe Virus Interferes With S-Acylation of Remorin and Induces Its Autophagic Degradation to Facilitate Virus Infection. Mol. Plant 11 (2), 269–287. doi: 10.1016/j.molp.2017.11.011 29229567

[B23] Galluzzi L. Green D. R. (2019). Autophagy-Independent Functions of the Autophagy Machinery. Cell 177 (7), 1682–1699. doi: 10.1016/j.cell.2019.05.026 31199916 PMC7173070

[B24] Gassen N. C. Papies J. Bajaj T. Emanuel J. Dethloff F. Chua R. L.. (2021). SARS-CoV-2-Mediated Dysregulation of Metabolism and Autophagy Uncovers Host-Targeting Antivirals. Nat. Commun. 12 (1), 3818. doi: 10.1038/s41467-021-24007-w 34155207 PMC8217552

[B25] Gatica D. Lahiri V. Klionsky D. J. (2018). Cargo Recognition and Degradation by Selective Autophagy. Nat. Cell Biol. 20 (3), 233–242. doi: 10.1038/s41556-018-0037-z 29476151 PMC6028034

[B26] Geng J. Klionsky D. J. (2008). The Atg8 and Atg12 Ubiquitin-Like Conjugation Systems in Macroautophagy. ’Protein Modifications: Beyond the Usual Suspects’ Review Series. EMBO Rep. 9 (9), 859–864. doi: 10.1038/embor.2008.163 18704115 PMC2529362

[B27] Hafren A. Macia J. L. Love A. J. Milner J. J. Drucker M. Hofius D. (2017). Selective Autophagy Limits Cauliflower Mosaic Virus Infection by NBR1-Mediated Targeting of Viral Capsid Protein and Particles. Proc. Natl. Acad. Sci. U.S.A. 114 (10), E2026–E2035. doi: 10.1073/pnas.1610687114 28223514 PMC5347569

[B28] Hafren A. Ustun S. Hochmuth A. Svenning S. Johansen T. Hofius D. (2018). Turnip Mosaic Virus Counteracts Selective Autophagy of the Viral Silencing Suppressor HCpro. Plant Physiol. 176 (1), 649–662. doi: 10.1104/pp.17.01198 29133371 PMC5761789

[B29] Haxim Y. Ismayil A. Jia Q. Wang Y. Zheng X. Chen T.. (2017). Autophagy Functions as an Antiviral Mechanism Against Geminiviruses in Plants. Elife 6, e23897. doi: 10.7554/eLife.23897 28244873 PMC5362266

[B30] Hayn M. Hirschenberger M. Koepke L. Nchioua R. Straub J. H. Klute S.. (2021). Systematic Functional Analysis of SARS-CoV-2 Proteins Uncovers Viral Innate Immune Antagonists and Remaining Vulnerabilities. Cell Rep. 35 (7):109126. doi: 10.1016/j.celrep.2021.109126 33974846 PMC8078906

[B31] He Y. Z. Wang Y. M. Yin T. Y. Fiallo-Olive E. Liu Y. Q. Hanley-Bowdoin L.. (2020). A Plant DNA Virus Replicates in the Salivary Glands of its Insect Vector. via recruitment Host DNA synth mach. Proc. Natl. Acad. Sci. U.S.A. 117 (29), 16928–16937. doi: 10.1073/pnas.1820132117 PMC7382290 32636269

[B32] Hosokawa N. Hara T. Kaizuka T. Kishi C. Takamura A. Miura Y.. (2009). Nutrient-Dependent Mtorc1 Association With the ULK1-Atg13-FIP200 Complex Required for Autophagy. Mol. Biol. Cell 20 (7), 1981–1991. doi: 10.1091/mbc.E08-12-1248 19211835 PMC2663915

[B33] Hou P. Yang K. Jia P. Liu L. Lin Y. Li Z.. (2021). A Novel Selective Autophagy Receptor, CCDC50, Delivers K63 Polyubiquitination-Activated RIG-I/MDA5 for Degradation During Viral Infection. Cell Res. 31 (1), 62–79. doi: 10.1038/s41422-020-0362-1 32612200 PMC7852694

[B34] Huang X. Chen S. Yang X. Yang X. Zhang T. Zhou G. (2020). Friend or Enemy: A Dual Role of Autophagy in Plant Virus Infection. Front. Microbiol. 11:736. doi: 10.3389/fmicb.2020.00736 32373106 PMC7186577

[B35] Hurley J. H. Young L. N. (2017). Mechanisms of Autophagy Initiation. Annu. Rev. Biochem. 86, 225–244. doi: 10.1146/annurev-biochem-061516-044820 28301741 PMC5604869

[B36] Ichimura Y. Kirisako T. Takao T. Satomi Y. Shimonishi Y. Ishihara N.. (2000). A Ubiquitin-Like System Mediates Protein Lipidation. Nature 408 (6811), 488–492. doi: 10.1038/35044114 11100732

[B37] Incarbone M. Dunoyer P. (2013). RNA Silencing and its Suppression: Novel Insights From in Planta Analyses. Trends Plant Sci. 18 (7), 382–392. doi: 10.1016/j.tplants.2013.04.001 23684690

[B38] Ismayil A. Yang M. Liu Y. (2020). Role of Autophagy During Plant-Virus Interactions. Semin. Cell Dev. Biol. 101, 36–40. doi: 10.1016/j.semcdb.2019.07.001 31291600

[B39] Itakura E. Kishi-Itakura C. Mizushima N. (2012). The Hairpin-Type Tail-Anchored SNARE Syntaxin 17 Targets to Autophagosomes for Fusion With Endosomes/Lysosomes. Cell 151 (6), 1256–1269. doi: 10.1016/j.cell.2012.11.001 23217709

[B40] Jia Q. Liu N. Xie K. Dai Y. Han S. Zhao X.. (2016). CLCuMuB Betac1 Subverts Ubiquitination by Interacting With NbSKP1s to Enhance Geminivirus Infection in Nicotiana Benthamiana. PloS Pathog. 12 (6), e1005668. doi: 10.1371/journal.ppat.1005668 27315204 PMC4912122

[B41] Jiang L. Lu Y. Zheng X. Yang X. Chen Y. Zhang T.. (2021). The Plant Protein NbP3IP Directs Degradation of Rice Stripe Virus P3 Silencing Suppressor Protein to Limit Virus Infection Through Interaction With the Autophagy-Related Protein Nbatg8. New Phytol. 229 (2), 1036–1051. doi: 10.1111/nph.16917 32898938

[B42] Johansen T. Lamark T. (2011). Selective Autophagy Mediated by Autophagic Adapter Proteins. Autophagy 7 (3), 279–296. doi: 10.4161/auto.7.3.14487 21189453 PMC3060413

[B43] Johansen T. Lamark T. (2020). Selective Autophagy: ATG8 Family Proteins, LIR Motifs and Cargo Receptors. J. Mol. Biol. 432 (1), 80–103. doi: 10.1016/j.jmb.2019.07.016 31310766

[B44] Jung H. Lee H. N. Marshall R. S. Lomax A. W. Yoon M. J. Kim J.. (2020). Arabidopsis Cargo Receptor NBR1 Mediates Selective Autophagy of Defective Proteins. J. Exp. Bot. 71 (1), 73–89. doi: 10.1093/jxb/erz404 31494674 PMC6913707

[B45] Kawai T. Akira S. (2011). Regulation of Innate Immune Signalling Pathways by the Tripartite Motif (TRIM) Family Proteins. EMBO Mol. Med. 3 (9), 513–527. doi: 10.1002/emmm.201100160 21826793 PMC3377094

[B46] Keller M. D. Torres V. J. Cadwell K. (2020). Autophagy and Microbial Pathogenesis. Cell Death Differ 27 (3), 872–886. doi: 10.1038/s41418-019-0481-8 31896796 PMC7205878

[B47] Khaminets A. Heinrich T. Mari M. Grumati P. Huebner A. K. Akutsu M.. (2015). Regulation of Endoplasmic Reticulum Turnover by Selective Autophagy. Nature 522 (7556), 354–358. doi: 10.1038/nature14498 26040720

[B48] Kim N. Kim M. J. Sung P. S. Bae Y. C. Shin E. C. Yoo J. Y. (2016). Interferon-Inducible Protein SCOTIN Interferes With HCV Replication Through the Autolysosomal Degradation of NS5A. Nat. Commun. 7:10631. doi: 10.1038/ncomms10631 26868272 PMC4754343

[B49] Kirkin V. Lamark T. Sou Y. S. Bjorkoy G. Nunn J. L. Bruun J. A.. (2009). A Role for NBR1 in Autophagosomal Degradation of Ubiquitinated Substrates. Mol. Cell 33 (4), 505–516. doi: 10.1016/j.molcel.2009.01.020 19250911

[B50] Koepke L. Hirschenberger M. Hayn M. Kirchhoff F. Sparrer K. M. (2021). Manipulation of Autophagy by SARS-CoV-2 Proteins. Autophagy 17 (9), 2659–2661. doi: 10.1080/15548627.2021.1953847 34281462 PMC8496524

[B51] Konrad S. S. Popp C. Stratil T. F. Jarsch I. K. Thallmair V. Folgmann J.. (2014). S-Acylation Anchors Remorin Proteins to the Plasma Membrane But Does Not Primarily Determine Their Localization in Membrane Microdomains. New Phytol. 203 (3), 758–769. doi: 10.1111/nph.12867 24897938

[B52] Kumar A. Singh R. Kaur J. Pandey S. Sharma V. Thakur L.. (2021). Wuhan to World: The COVID-19 Pandemic. Front. Cell Infect. Microbiol. 11:596201. doi: 10.3389/fcimb.2021.596201 33859951 PMC8042280

[B53] Kushwaha N. K. Hafren A. Hofius D. (2019). Autophagy-Virus Interplay in Plants: From Antiviral Recognition to Proviral Manipulation. Mol. Plant Pathol. 20 (9), 1211–1216. doi: 10.1111/mpp.12852 31397085 PMC6715616

[B54] Ku B. Woo J. S. Liang C. Lee K. H. Hong H. S, E, X.. (2008). Structural and Biochemical Bases for the Inhibition of Autophagy and Apoptosis by Viral BCL-2 of Murine Gamma-Herpesvirus 68. PloS Pathog. 4 (2), e25. doi: 10.1371/journal.ppat.0040025 18248095 PMC2222952

[B55] Leary A. Y. Savage Z. Tumtas Y. Bozkurt T. O. (2019). Contrasting and Emerging Roles of Autophagy in Plant Immunity. Curr. Opin. Plant Biol. 52, 46–53. doi: 10.1016/j.pbi.2019.07.002 31442734

[B56] Lee J. S. Li Q. Lee J. Y. Lee S. H. Jeong J. H. Lee H. R.. (2009). FLIP-Mediated Autophagy Regulation in Cell Death Control. Nat. Cell Biol. 11 (11), 1355–1362. doi: 10.1038/ncb1980 19838173 PMC2802862

[B57] Lennemann N. J. Coyne C. B. (2017). Dengue and Zika Viruses Subvert Reticulophagy by NS2B3-Mediated Cleavage of FAM134B. Autophagy 13 (2), 322–332. doi: 10.1080/15548627.2016.1265192 28102736 PMC5324851

[B58] Levine B. Liu R. Dong X. Zhong Q. (2015). Beclin Orthologs: Integrative Hubs of Cell Signaling, Membrane Trafficking, and Physiology. Trends Cell Biol. 25 (9), 533–544. doi: 10.1016/j.tcb.2015.05.004 26071895 PMC4554927

[B59] Li F. Huang C. Li Z. Zhou X. (2014). Suppression of RNA Silencing by a Plant DNA Virus Satellite Requires a Host Calmodulin-Like Protein to Repress RDR6 Expression. PloS Pathog. 10 (2), e1003921. doi: 10.1371/journal.ppat.1003921 24516387 PMC3916407

[B60] Li Y. Hu B. Ji G. Zhang Y. Xu C. Lei J.. (2020c). Cytoplasmic Cargo Receptor P62 Inhibits Avibirnavirus Replication by Mediating Autophagic Degradation of Viral Protein Vp2. J. Virol. 94 (24), e01255-20. doi: 10.1128/JVI.01255-20 32967959 PMC7925189

[B61] Li J. Liu Y. Wang Z. Liu K. Wang Y. Liu J.. (2011). Subversion of Cellular Autophagy Machinery by Hepatitis B Virus for Viral Envelopment. J. Virol. 85 (13), 6319–6333. doi: 10.1128/JVI.02627-10 21507968 PMC3126540

[B62] Liu B. Fang M. Hu Y. Huang B. Li N. Chang C.. (2014). Hepatitis B Virus X Protein Inhibits Autophagic Degradation by Impairing Lysosomal Maturation. Autophagy 10 (3), 416–430. doi: 10.4161/auto.27286 24401568 PMC4077881

[B63] Liu S. Mok B. W. Deng S. Liu H. Wang P. Song W.. (2021). Mammalian Cells Use the Autophagy Process to Restrict Avian Influenza Virus Replication. Cell Rep. 35 (10):109213. doi: 10.1016/j.celrep.2021.109213 34107256

[B64] Liu Y. Schiff M. Czymmek K. Talloczy Z. Levine B. Dinesh-Kumar S. P. (2005). Autophagy Regulates Programmed Cell Death During the Plant Innate Immune Response. Cell 121 (4), 567–577. doi: 10.1016/j.cell.2005.03.007 15907470

[B65] Li C. Xu Y. Fu S. Liu Y. Li Z. Zhang T.. (2021). The Unfolded Protein Response Plays Dual Roles in Rice Stripe Virus Infection Through Fine-Tuning the Movement Protein Accumulation. PloS Pathog. 17 (3), e1009370. doi: 10.1371/journal.ppat.1009370 33662041 PMC8075255

[B66] Li F. Zhang C. Li Y. Wu G. Hou X. Zhou X.. (2018). Beclin1 Restricts RNA Virus Infection in Plants Through Suppression and Degradation of the Viral Polymerase. Nat. Commun. 9 (1), 1268. doi: 10.1038/s41467-018-03658-2 29593293 PMC5871769

[B67] Li F. Zhang C. Tang Z. Zhang L. Dai Z. Lyu S.. (2020a). A Plant RNA Virus Activates Selective Autophagy in a UPR-Dependent Manner to Promote Virus Infection. New Phytol. 228 (2), 622–639. doi: 10.1111/nph.16716 32479643

[B68] Li F. Zhang M. Zhang C. Zhou X. (2020b). Nuclear Autophagy Degrades a Geminivirus Nuclear Protein to Restrict Viral Infection in Solanaceous Plants. New Phytol. 225 (4), 1746–1761. doi: 10.1111/nph.16268 31621924

[B69] Li F. Zhao N. Li Z. Xu X. Wang Y. Yang X.. (2017). A Calmodulin-Like Protein Suppresses RNA Silencing and Promotes Geminivirus Infection by Degrading Sgs3. via autophagy pathway Nicotiana benthamiana. PloS Pathog. 13 (2), e1006213. doi: 10.1371/journal.ppat.1006213 28212430 PMC5333915

[B70] Lussignol M. Esclatine A. (2017). Herpesvirus and Autophagy: “All Right, Everybody Be Cool, This Is a Robbery!”. Viruses 9 (12), 372. doi: 10.3390/v9120372 PMC5744147 29207540

[B71] Mandadi K. K. Scholthof K. B. (2013). Plant Immune Responses Against Viruses: How Does a Virus Cause Disease? Plant Cell 25 (5), 1489–1505. doi: 10.1105/tpc.113.111658 23709626 PMC3694688

[B72] Mandell M. A. Jain A. Arko-Mensah J. Chauhan S. Kimura T. Dinkins C.. (2014a). TRIM Proteins Regulate Autophagy and can Target Autophagic Substrates by Direct Recognition. Dev. Cell 30 (4), 394–409. doi: 10.1016/j.devcel.2014.06.013 25127057 PMC4146662

[B73] Mandell M. A. Kimura T. Jain A. Johansen T. Deretic V. (2014b). TRIM Proteins Regulate Autophagy: TRIM5 is a Selective Autophagy Receptor Mediating HIV-1 Restriction. Autophagy 10 (12), 2387–2388. doi: 10.4161/15548627.2014.984278 25587751 PMC4502693

[B74] Marshall R. S. Hua Z. Mali S. McLoughlin F. Vierstra R. D. (2019). ATG8-Binding UIM Proteins Define a New Class of Autophagy Adaptors and Receptors. Cell 177 (3), 766–781 e724. doi: 10.1016/j.cell.2019.02.009 30955882 PMC6810650

[B75] Marshall R. S. Vierstra R. D. (2018). Autophagy: The Master of Bulk and Selective Recycling. Annu. Rev. Plant Biol. 69, 173–208. doi: 10.1146/annurev-arplant-042817-040606 29539270

[B76] Martin-Sancho L. Tripathi S. Rodriguez-Frandsen A. Pache L. Sanchez-Aparicio M. McGregor M. J.. (2021). Restriction Factor Compendium for Influenza A Virus Reveals a Mechanism for Evasion of Autophagy. Nat. Microbiol. 6 (10), 1319–1333. doi: 10.1038/s41564-021-00964-2 34556855 PMC9683089

[B77] Miao G. Zhao H. Li Y. Ji M. Chen Y. Shi Y.. (2021). ORF3a of the COVID-19 Virus SARS-CoV-2 Blocks HOPS Complex-Mediated Assembly of the SNARE Complex Required for Autolysosome Formation. Dev. Cell 56 427-442 (4), e425. doi: 10.1016/j.devcel.2020.12.010 PMC7832235 33422265

[B78] Michaeli S. Clavel M. Lechner E. Viotti C. Wu J. Dubois M.. (2019). The Viral F-Box Protein P0 Induces an ER-Derived Autophagy Degradation Pathway for the Clearance of Membrane-Bound Ago1. Proc. Natl. Acad. Sci. U.S.A. 116 (45), 22872–22883. doi: 10.1073/pnas.1912222116 31628252 PMC6842623

[B79] Michaeli S. Galili G. Genschik P. Fernie A. R. Avin-Wittenberg T. (2016). Autophagy in Plants–What’s New on the Menu? Trends Plant Sci. 21 (2), 134–144. doi: 10.1016/j.tplants.2015.10.008 26598298

[B80] Mizushima N. Komatsu M. (2011). Autophagy: Renovation of Cells and Tissues. Cell 147 (4), 728–741. doi: 10.1016/j.cell.2011.10.026 22078875

[B81] Mohamud Y. Qu J. Xue Y. C. Liu H. Deng H. Luo H. (2019). CALCOCO2/NDP52 and SQSTM1/p62 Differentially Regulate Coxsackievirus B3 Propagation. Cell Death Differ 26 (6), 1062–1076. doi: 10.1038/s41418-018-0185-5 30154446 PMC6748094

[B82] Mouna L. Hernandez E. Bonte D. Brost R. Amazit L. Delgui L. R.. (2016). Analysis of the Role of Autophagy Inhibition by Two Complementary Human Cytomegalovirus BECN1/Beclin 1-Binding Proteins. Autophagy 12 (2), 327–342. doi: 10.1080/15548627.2015.1125071 26654401 PMC4836022

[B83] Mukherjee S. (2020). Before Virus, After Virus: A Reckoning. Cell 183 (2), 308–314. doi: 10.1016/j.cell.2020.09.042 33064987 PMC7560376

[B84] Muscolino E. Schmitz R. Loroch S. Caragliano E. Schneider C. Rizzato M.. (2020). Herpesviruses Induce Aggregation and Selective Autophagy of Host Signalling Proteins NEMO and RIPK1 as an Immune-Evasion Mechanism. Nat. Microbiol. 5 (2), 331–342. doi: 10.1038/s41564-019-0624-1 31844296

[B85] Nakahara K. S. Masuta C. Yamada S. Shimura H. Kashihara Y. Wada T. S.. (2012). Tobacco Calmodulin-Like Protein Provides Secondary Defense by Binding to and Directing Degradation of Virus RNA Silencing Suppressors. Proc. Natl. Acad. Sci. U.S.A. 109 (25), 10113–10118. doi: 10.1073/pnas.1201628109 22665793 PMC3382489

[B86] Nakatogawa H. (2013). Two Ubiquitin-Like Conjugation Systems That Mediate Membrane Formation During Autophagy. Essays Biochem. 55, 39–50. doi: 10.1042/bse0550039 24070470

[B87] Nakatogawa H. Ichimura Y. Ohsumi Y. (2007). Atg8, a Ubiquitin-Like Protein Required for Autophagosome Formation, Mediates Membrane Tethering and Hemifusion. Cell 130 (1), 165–178. doi: 10.1016/j.cell.2007.05.021 17632063

[B88] Nicaise V. (2014). Crop Immunity Against Viruses: Outcomes and Future Challenges. Front. Plant Sci. 5:660. doi: 10.3389/fpls.2014.00660 25484888 PMC4240047

[B89] Orvedahl A. Alexander D. Talloczy Z. Sun Q. Wei Y. Zhang W.. (2007). HSV-1 ICP34.5 Confers Neurovirulence by Targeting the Beclin 1 Autophagy Protein. Cell Host Microbe 1 (1), 23–35. doi: 10.1016/j.chom.2006.12.001 18005679

[B90] Orvedahl A. MacPherson S. Sumpter R. Jr. Talloczy Z. Zou Z. Levine B. (2010). Autophagy Protects Against Sindbis Virus Infection of the Central Nervous System. Cell Host Microbe 7 (2), 115–127. doi: 10.1016/j.chom.2010.01.007 20159618 PMC2860265

[B91] Orvedahl A. Sumpter R. Jr. Xiao G. Ng A. Zou Z. Tang Y.. (2011). Image-Based Genome-Wide siRNA Screen Identifies Selective Autophagy Factors. Nature 480 (7375), 113–117. doi: 10.1038/nature10546 22020285 PMC3229641

[B92] Pattingre S. Tassa A. Qu X. Garuti R. Liang X. H. Mizushima N.. (2005). Bcl-2 Antiapoptotic Proteins Inhibit Beclin 1-Dependent Autophagy. Cell 122 (6), 927–939. doi: 10.1016/j.cell.2005.07.002 16179260

[B93] Paul P. Munz C. (2016). Autophagy and Mammalian Viruses: Roles in Immune Response, Viral Replication, and Beyond. Adv. Virus Res. 95, 149–195. doi: 10.1016/bs.aivir.2016.02.002 27112282

[B94] Polson H. E. de Lartigue J. Rigden D. J. Reedijk M. Urbe S. Clague M. J.. (2010). Mammalian Atg18 (WIPI2) Localizes to Omegasome-Anchored Phagophores and Positively Regulates LC3 Lipidation. Autophagy 6 (4), 506–522. doi: 10.4161/auto.6.4.11863 20505359

[B95] Proikas-Cezanne T. Takacs Z. Donnes P. Kohlbacher O. (2015). WIPI Proteins: Essential PtdIns3P Effectors at the Nascent Autophagosome. J. Cell Sci. 128 (2), 207–217. doi: 10.1242/jcs.146258 25568150

[B96] Pu Y. Luo X. Bassham D. C. (2017). TOR-Dependent and -Independent Pathways Regulate Autophagy in Arabidopsis Thaliana. Front. Plant Sci. 8:1204. doi: 10.3389/fpls.2017.01204 28744293 PMC5504165

[B97] Qu Y. Wang X. Zhu Y. Wang W. Wang Y. Hu G.. (2021). ORF3a-Mediated Incomplete Autophagy Facilitates Severe Acute Respiratory Syndrome Coronavirus-2 Replication. Front. Cell Dev. Biol. 9:716208. doi: 10.3389/fcell.2021.716208 34386498 PMC8353283

[B98] Ran J. Hashimi S. M. Liu J. Z. (2020). Emerging Roles of the Selective Autophagy in Plant Immunity and Stress Tolerance. Int. J. Mol. Sci. 21 (17), 6321. doi: 10.3390/ijms21176321 PMC7503401 32878263

[B99] Ribeiro C. M. Sarrami-Forooshani R. Setiawan L. C. Zijlstra-Willems E. M. van Hamme J. L. Tigchelaar W.. (2016). Receptor Usage Dictates HIV-1 Restriction by Human TRIM5alpha in Dendritic Cell Subsets. Nature 540 (7633), 448–452. doi: 10.1038/nature20567 27919079

[B100] Rubio R. M. Mohr I. (2019). Inhibition of ULK1 and Beclin1 by an Alpha-Herpesvirus Akt-Like Ser/Thr Kinase Limits Autophagy to Stimulate Virus Replication. Proc. Natl. Acad. Sci. U.S.A. 116 (52), 26941–26950. doi: 10.1073/pnas.1915139116 PMC6936557 31843932

[B101] Russell R. C. Tian Y. Yuan H. Park H. W. Chang Y. Y. Kim J.. (2013). ULK1 Induces Autophagy by Phosphorylating Beclin-1 and Activating VPS34 Lipid Kinase. Nat. Cell Biol. 15 (7), 741–750. doi: 10.1038/ncb2757 23685627 PMC3885611

[B102] Sagnier S. Daussy C. F. Borel S. Robert-Hebmann V. Faure M. Blanchet F. P.. (2015). Autophagy Restricts HIV-1 Infection by Selectively Degrading Tat in CD4+ T Lymphocytes. J. Virol. 89 (1), 615–625. doi: 10.1128/JVI.02174-14 25339774 PMC4301118

[B103] Shi J. Wong J. Piesik P. Fung G. Zhang J. Jagdeo J.. (2013). Cleavage of Sequestosome 1/P62 by an Enteroviral Protease Results in Disrupted Selective Autophagy and Impaired NFKB Signaling. Autophagy 9 (10), 1591–1603. doi: 10.4161/auto.26059 23989536

[B104] Sir D. Chen W. L. Choi J. Wakita T. Yen T. S. Ou J. H. (2008). Induction of Incomplete Autophagic Response by Hepatitis C Virus via the Unfolded Protein Response. Hepatology 48 (4), 1054–1061. doi: 10.1002/hep.22464 18688877 PMC2562598

[B105] Sir D. Tian Y. Chen W. L. Ann D. K. Yen T. S. Ou J. H. (2010). The Early Autophagic Pathway is Activated by Hepatitis B Virus and Required for Viral DNA Replication. Proc. Natl. Acad. Sci. U.S.A. 107 (9), 4383–4388. doi: 10.1073/pnas.0911373107 20142477 PMC2840127

[B106] Soto-Burgos J. Zhuang X. Jiang L. Bassham D. C. (2018). Dynamics of Autophagosome Formation. Plant Physiol. 176 (1), 219–229. doi: 10.1104/pp.17.01236 29061903 PMC5761814

[B107] Staring J. von Castelmur E. Blomen V. A. van den Hengel L. G. Brockmann M. Baggen J.. (2017). PLA2G16 Represents a Switch Between Entry and Clearance of Picornaviridae. Nature 541 (7637), 412–416. doi: 10.1038/nature21032 28077878

[B108] Sumpter R. Jr. Sirasanagandla S. Fernandez A. F. Wei Y. Dong X. Franco L.. (2016). Fanconi Anemia Proteins Function in Mitophagy and Immunity. Cell 165 (4), 867–881. doi: 10.1016/j.cell.2016.04.006 27133164 PMC4881391

[B109] Taubenberger J. K. Kash J. C. (2010). Influenza Virus Evolution, Host Adaptation, and Pandemic Formation. Cell Host Microbe 7 (6), 440–451. doi: 10.1016/j.chom.2010.05.009 20542248 PMC2892379

[B110] Tong X. Liu S. Y. Zou J. Z. Zhao J. J. Zhu F. F. Chai L. X.. (2021). A Small Peptide Inhibits siRNA Amplification in Plants by Mediating Autophagic Degradation of SGS3/RDR6 Bodies. EMBO J. 40 (15), e108050. doi: 10.15252/embj.2021108050 34155657 PMC8327956

[B111] V’Kovski P. Kratzel A. Steiner S. Stalder H. Thiel V. (2021). Coronavirus Biology and Replication: Implications for SARS-CoV-2. Nat. Rev. Microbiol. 19 (3), 155–170. doi: 10.1038/s41579-020-00468-6 33116300 PMC7592455

[B112] Vietri M. Radulovic M. Stenmark H. (2020). The Many Functions of ESCRTs. Nat. Rev. Mol. Cell Biol. 21 (1), 25–42. doi: 10.1038/s41580-019-0177-4 31705132

[B113] Vo M. T. Choi Y. B. (2021). Herpesvirus Regulation of Selective Autophagy. Viruses 13 (5), 820. doi: 10.3390/v13050820 34062931 PMC8147283

[B114] Wang Y. Sharma P. Jefferson M. Zhang W. Bone B. Kipar A.. (2021b). Non-Canonical Autophagy Functions of ATG16L1 in Epithelial Cells Limit Lethal Infection by Influenza A Virus. EMBO J. 40 (6), e105543. doi: 10.15252/embj.2020105543 33586810 PMC7957399

[B115] Wang R. Zhu Y. Ren C. Yang S. Tian S. Chen H.. (2021a). Influenza A Virus Protein PB1-F2 Impairs Innate Immunity by Inducing Mitophagy. Autophagy 17 (2), 496–511. doi: 10.1080/15548627.2020.1725375 32013669 PMC8007153

[B116] Wang R. Zhu Y. Zhao J. Ren C. Li P. Chen H.. (2019). Autophagy Promotes Replication of Influenza A Virus. In Vitro. J. Virol. 93 (4), e01984-18. doi: 10.1128/JVI.01984-18 30541828 PMC6363991

[B117] Wei Y. Sinha S. Levine B. (2008). Dual Role of JNK1-Mediated Phosphorylation of Bcl-2 in Autophagy and Apoptosis Regulation. Autophagy 4 (7), 949–951. doi: 10.4161/auto.6788 18769111 PMC2677707

[B118] Welsch S. Miller S. Romero-Brey I. Merz A. Bleck C. K. Walther P.. (2009). Composition and Three-Dimensional Architecture of the Dengue Virus Replication and Assembly Sites. Cell Host Microbe 5 (4), 365–375. doi: 10.1016/j.chom.2009.03.007 19380115 PMC7103389

[B119] Xie Z. Klionsky D. J. (2007). Autophagosome Formation: Core Machinery and Adaptations. Nat. Cell Biol. 9 (10), 1102–1109. doi: 10.1038/ncb1007-1102 17909521

[B120] Yang M. Ismayil A. Liu Y. (2020). Autophagy in Plant-Virus Interactions. Annu. Rev. Virol. 7 (1), 403–419. doi: 10.1146/annurev-virology-010220-054709 32530794

[B121] Yang F. Kimberlin A. N. Elowsky C. G. Liu Y. Gonzalez-Solis A. Cahoon E. B.. (2019). A Plant Immune Receptor Degraded by Selective Autophagy. Mol. Plant 12 (1), 113–123. doi: 10.1016/j.molp.2018.11.011 30508598

[B122] Yang M. Zhang Y. Xie X. Yue N. Li J. Wang X. B.. (2018). Barley Stripe Mosaic Virus Gammab Protein Subverts Autophagy to Promote Viral Infection by Disrupting the ATG7-ATG8 Interaction. Plant Cell 30 (7), 1582–1595. doi: 10.1105/tpc.18.00122 29848767 PMC6096602

[B123] Yim W. W. Mizushima N. (2021). Autophagosome Maturation Stymied by SARS-CoV-2. Dev. Cell 56 (4), 400–402. doi: 10.1016/j.devcel.2021.02.002 33621488 PMC7898975

[B124] Yla-Anttila P. (2021). Autophagy Receptors as Viral Targets. Cell Mol. Biol. Lett. 26 (1), 29. doi: 10.1186/s11658-021-00272-x 34167456 PMC8222950

[B125] Yla-Anttila P. Gupta S. Masucci M. G. (2021). The Epstein-Barr Virus Deubiquitinase BPLF1 Targets SQSTM1/p62 to Inhibit Selective Autophagy. Autophagy 17 (11), 1–15. doi: 10.1080/15548627.2021.1874660 33509017 PMC8632276

[B126] Yordy B. Iijima N. Huttner A. Leib D. Iwasaki A. (2012). A Neuron-Specific Role for Autophagy in Antiviral Defense Against Herpes Simplex Virus. Cell Host Microbe 12 (3), 334–345. doi: 10.1016/j.chom.2012.07.013 22980330 PMC3454454

[B127] Yordy B. Tal M. C. Hayashi K. Arojo O. Iwasaki A. (2013). Autophagy and Selective Deployment of Atg Proteins in Antiviral Defense. Int. Immunol. 25 (1), 1–10. doi: 10.1093/intimm/dxs101 23042773 PMC3534236

[B128] Young A. R. Chan E. Y. Hu X. W. Kochl R. Crawshaw S. G. High S.. (2006). Starvation and ULK1-Dependent Cycling of Mammalian Atg9 Between the TGN and Endosomes. J. Cell Sci. 119 (Pt 18), 3888–3900. doi: 10.1242/jcs.03172 16940348

[B129] Zheng Y. Zhu G. Tang Y. Yan J. Han S. Yin J.. (2020). HDAC6, A Novel Cargo for Autophagic Clearance of Stress Granules, Mediates the Repression of the Type I Interferon Response During Coxsackievirus A16 Infection. Front. Microbiol. 11:78. doi: 10.3389/fmicb.2020.00078 32082291 PMC7005486

[B130] Zhuang X. Chung K. P. Cui Y. Lin W. Gao C. Kang B. H.. (2017). ATG9 Regulates Autophagosome Progression From the Endoplasmic Reticulum in Arabidopsis. Proc. Natl. Acad. Sci. U.S.A. 114 (3), E426–E435. doi: 10.1073/pnas.1616299114 28053229 PMC5255614

[B131] Zvereva A. S. Golyaev V. Turco S. Gubaeva E. G. Rajeswaran R. Schepetilnikov M. V.. (2016). Viral Protein Suppresses Oxidative Burst and Salicylic Acid-Dependent Autophagy and Facilitates Bacterial Growth on Virus-Infected Plants. New Phytol. 211 (3), 1020–1034. doi: 10.1111/nph.13967 27120694

